# Telomeric-Like Repeats Flanked by Sequences Retrotranscribed from the Telomerase RNA Inserted at DNA Double-Strand Break Sites during Vertebrate Genome Evolution

**DOI:** 10.3390/ijms222011048

**Published:** 2021-10-13

**Authors:** Lorenzo Sola, Solomon G. Nergadze, Eleonora Cappelletti, Francesca M. Piras, Elena Giulotto, Marco Santagostino

**Affiliations:** Department of Biology and Biotechnology “L. Spallanzani”, University of Pavia, 27100 Pavia, Italy; lorenzo.sola01@universitadipavia.it (L.S.); solomon.nergadze@unipv.it (S.G.N.); eleonora.cappelletti01@universitadipavia.it (E.C.); mfrancesca.piras@unipv.it (F.M.P.); marco.santagostino@unipv.it (M.S.)

**Keywords:** repetitive DNA sequences, telomerase, telomerase RNA, telomeric repeats, interstitial telomeres, genome evolution, vertebrates, DNA repair

## Abstract

Interstitial telomeric sequences (ITSs) are stretches of telomeric-like repeats located at internal chromosomal sites. We previously demonstrated that ITSs have been inserted during the repair of DNA double-strand breaks in the course of evolution and that some rodent ITSs, called TERC-ITSs, are flanked by fragments retrotranscribed from the telomerase RNA component (TERC). In this work, we carried out an extensive search of TERC-ITSs in 30 vertebrate genomes and identified 41 such loci in 22 species, including in humans and other primates. The fragment retrotranscribed from the TERC RNA varies in different lineages and its sequence seems to be related to the organization of TERC. Through comparative analysis of TERC-ITSs with orthologous empty loci, we demonstrated that, at each locus, the TERC-like sequence and the ITS have been inserted in one step in the course of evolution. Our findings suggest that telomerase participated in a peculiar pathway of DNA double-strand break repair involving retrotranscription of its RNA component and that this mechanism may be active in all vertebrate species. These results add new evidence to the hypothesis that RNA-templated DNA repair mechanisms are active in vertebrate cells.

## 1. Introduction

Telomeres are specialized nucleoprotein structures located at the termini of linear chromosomes. The main function of telomeres is to protect the ends of chromosomes by differentiating them from DNA double-strand breaks [1]. In vertebrates, telomeres are composed of tandem arrays of the TTAGGG hexanucleotide which are associated with a protein complex called shelterin and with other accessory proteins [1]. Telomeres are transcribed into a family of noncoding telomeric repeat-containing RNA (TERRA) molecules [2]. The association of telomeric DNA with proteins and TERRA is essential for the maintenance of telomere structure and function [3]. Deregulation of TERRA expression has also been proposed as a marker for tumor progression [4,5].

In normal somatic cells, telomeres progressively shorten at each replication cycle while, in germ line and stem cells, they are maintained by the enzyme telomerase, a specialized nucleoprotein that adds telomeric repeats to the 3′ end of telomeric DNA. Telomerase is composed of a protein with reverse transcriptase activity (telomerase reverse transcriptase, TERT), and an RNA molecule (telomerase RNA component, TERC) containing the template for the synthesis of telomeric repeats. The length of TERC RNA is variable, ranging from a few hundred nucleotides (140–210 nt) in ciliates to a few thousand (900–2452 nt) in fungi. In the vertebrates in which TERC RNA has been characterized, its size ranges from 312 to 559 nt [6,7,8,9]. Together with size, the sequence and secondary structure of TERC also vary among Eukarya. The approach for TERC identification, based on the biochemical purification of the telomerase holoenzyme [10,11], is often challenging. Alternatively, PCR-based approaches and bioinformatic tools, such as BLAST, have been successfully utilized to identify the *TERC* gene in evolutionarily related species [6,7,11,12]. In vertebrates, TERC possesses a conserved secondary structure comprising a pseudoknot, containing the telomeric repeat template at the 5′ end, and three partial stem loops required for TERC precursor maturation and interaction with TERT [13,14,15,16,17,18]. 

The telomerase enzyme adds telomeric repeats to telomeres through reverse transcription of the template located in the RNA moiety. Multiple cycles of template-telomere annealing, retrotranscription of the template, and translocation to a new position on the substrate result in the addition of a telomeric repeat array [19]. 

Stretches of telomeric-like repeats positioned at non-terminal sites of chromosomes are called interstitial telomeric sequences (ITSs) [20]. Unlike telomeres, whose role in genome stability is well known, the function of ITSs still needs to be defined. ITSs have been classified according to chromosomal localization and sequence organization [21]. Heterochromatic ITSs are extended blocks of telomeric-like repeats mainly localized at pericentromeres or subtelomeres [22,23,24,25,26,27] and are easily identified by FISH. Fusion ITSs are generated by telomere–telomere fusion and are organized in a head-to-head fashion [28,29]. Short ITSs (s-ITSs) are arrays of a few to a few hundred exact or almost exact telomeric repeats randomly distributed at internal chromosomal sites. Given the limited number of repeats at these loci, their detection by FISH, using telomeric probes, is inefficient and they have mainly been identified through genome sequence analysis. We previously studied s-ITSs in primates [29,30,31,32,33,34], rodents [34], and perissodactyls [35]. S-ITS can be considered a particular type of microsatellite composed of the repetition of 6 bp long units. Whereas canonical microsatellites are generated via DNA polymerase slippage that causes the expansion of pre-existing units, s-ITSs originated from a totally different mechanism [32,34]. Through comparative analysis of human s-ITS-containing loci and the corresponding ITS-less orthologs from non-human primates, we showed that s-ITSs were introduced in one step during evolution. Following these findings, we proposed that s-ITSs were inserted in genomes through a peculiar pathway of DNA double-strand break repair [32]. Our work on ITS loci in rodents [34] and equids [35] provided further evidence for this hypothesis. We then tested whether s-ITSs can be inserted at DNA double-strand breaks in cultured somatic cells by inducing site-specific breaks catalyzed by the enzyme I-SceI [36]. Although we analyzed more than 35,000 junctions generated by the repair of DNA break, we never observed breaks repaired through the insertion of ITSs, suggesting that, in this experimental system, such events are rare or absent. Using a similar experimental set up, Onozawa and colleagues transfected cultured human cancer cells with total cellular RNA and showed that DNA double-strand breaks can be repaired through the introduction of sequences reverse transcribed from the exogenous RNA [37]. Interestingly, these authors found that telomeric repeats were inserted at three break sites.

TERC-ITSs are s-ITSs characterized by a peculiar sequence organization that we identified in mouse and rat genomes [34]. At these loci, the telomeric repeat stretch is flanked by a sequence reverse transcribed from the 3′ domain of TERC [34]. We recently identified a TERC-ITS locus in the horse genome which contains a sequence derived from the pseudoknot [35]. The peculiar organization of these loci strongly suggested that the telomerase enzyme may be involved in their insertion of ITSs in genomes.

Finally, strong evidence supports the hypothesis that s-ITSs and TERC-ITSs were inserted in genomes during the repair of DNA double strand breaks: (i) they appeared suddenly during evolution at unrelated loci [29,31,32,34,35] and (ii) their insertion is usually accompanied by modifications of the insertion sites typical of the non-homologous end-joining repair pathway, such as deletions and random sequence addition [29,31,32,34,35,36,37].

In the present work, we carried out an extensive search of TERC-ITSs and found that such loci are present throughout the vertebrate evolutionary tree. We then dated their origin during evolution and analyzed their sequence organization, showing evidence that the structure of TERC may influence the choice of the fragments to be retrotranscribed at TERC-ITS loci. Finally, we proposed new models for the generation of TERC-ITSs.

## 2. Results

### 2.1. The Search for TERC-ITS Loci in Vertebrates

In our previous work, we demonstrated that, in the mouse and rat genome, the generation of TERC-ITSs occurred during the repair of DNA double-strand breaks and involved the insertion of fragments retrotranscribed from the telomerase RNA [34,35]. 

In the present work, we searched for TERC-ITS loci in the genome of 30 vertebrate species that were selected from different orders on the basis of the availability of well assembled genomic sequences and an identifiable *TERC* gene. In particular, we searched for TERC-ITSs in eight species of the order Primates (*Homo sapiens,* human, HSA; *Pan troglodytes*, common chimpanzee, PTR; *Gorilla gorilla gorilla*, western lowland gorilla, GGG; *Pongo pygmaeus abelii*, Sumatran orangutan, PPA; *Macaca mulatta*, rhesus macaque, MMU; *Callithrix jacchus*, common marmoset, CJA; *Saimiri boliviensis,* black-capped squirrel monkey, SBO; *Microcebus murinus*, gray mouse lemur, MMR), one from Lagomorpha (*Oryctolagus cuniculus*, European rabbit, OCU), two from Carnivora (*Canis lupus familiaris*, domestic dog, CLF; *Felis catus*, domestic cat, FCA), three from Rodentia (*Mus musculus musculus*, mouse, Mus; *Rattus norvegicus*, rat, RNO; *Cricetulus griseus*, Chinese hamster, CGR), two from Artiodactyla (*Bos taurus*, cow, BTA; *Sus scrofa*, pig, SSC), two from Perissodactyla (*Equus caballus*, horse, ECA; *Tapirus indicus*, Malayan tapir, TIN), one from Proboscidea (*Loxodonta africana*, African bush elephant, LAF), one from Diprotodontia (*Macropus eugenii*, tammar wallaby, MEU), one from Dasyuromorphia (*Sarcophilus harrisii*, Tasmanian devil, SHA), one from Galliformes (*Gallus gallus*, red junglefowl, GGA), one from Passeriformes (*Hirundo rustica*, barn swallow, HRU), one from Psittaciformes (*Anodorhynchus hyacinthinus*, Hyacinth macaw, AHY), one from Anura (*Xenopus laevis,* African clawed frog, XLA), one from Tetraodontiformes (*Takifugu rubripes*, tiger puffer, TRU), one from Carcharhiniformes (*Scyliorhinus torazame*, cloudy catshark, STO), two from Petromyzontiformes (*Petromyzon marinus*, sea lamprey, PMA; *Lethenteron camtschaticum*, arctic lamprey, LCA), and one from Myxiniformes (*Eptatretus burgeri*, Inshore hagfish, EBU). For most species, we retrieved the sequence of the *TERC* gene from either the NCBI Gene Database (https://www.ncbi.nlm.nih.gov/gene) [38] or the Telomerase Database (http://telomerase.asu.edu) [39,40], whereas, for other species, we BLAST-searched the putative *TERC* gene utilizing the TERC sequence of evolutionarily related species as the query (as specified in Section 4). In Table S1, all *TERC* gene sequences used in this study are reported. We then used the *TERC* gene sequence identified in each species as a query to BLAST-search TERC-like insertions in the genome assembly. Sequences adjacent to each BLAST hit were then scanned for the presence of telomeric hexamers. In our previous work, we searched for stretches composed of at least four telomeric repeats. In this work, we used less stringent parameters and included loci comprising at least one telomeric hexamer adjacent to a TERC-like sequence. Using this method, we detected 41 TERC-ITS loci (Table 1), some of which are conserved in evolutionarily related species (Table 1 and Table S2). Altogether, TERC-ITS loci were found in 22 species (HSA, PTR, GGG, PPA, MMU, CJA, SBO, MMR, CGR, Mus, RNO, OCU, TIN, BTA, ECA, SSC, LAF, MEU, HRU, XLA, TRU, and STO) (Table 1). In the remaining eight species (CLF, FCA, SHA, AHY, GGA, PMA, LCA, and EBU), no TERC-ITS loci were found. In our previous work [34], we did not find any TERC-ITS in primates. In the present study, thanks to the new search parameters, we found two loci in the human genome (locus number 1 and 2) that are conserved in all informative Simiiformes primates. In addition, five TERC-ITSs were found in the lemur *Microcebus murinus*. In *Bos taurus*, *Oryctolagus cuniculus*, and in the shark *Scyliorhinus torazame*, two TERC-ITS loci were found whereas in all other species only one locus was detected.

In our previous work, we identified 14 TERC-ITS loci in mice, 3 in rats [34], and 1 in horses [35]. In the present work, no additional horse loci were detected, whereas two additional TERC-ITSs were found in the mouse genome, one of which is conserved in the rat. These new loci are listed in Table 1 together with those described in our previous analysis. Here we extended the analysis to another species from the Rodentia order (*Cricetulus griseus*), where we found four new TERC-ITSs, and to another species from Perissodactyla (*Tapirus indicus*), where we found one new TERC-ITS locus.

Altogether, our search showed that TERC-ITSs are present throughout the entire phylogenetic tree of Vertebrata and that this type of insertion is particularly represented in Rodentia.

It is worth mentioning that, as previously reported by our group for mice [34], TERC pseudogenes, not flanked by telomeric hexamers, have been identified in pigs (SSC1:57646783), cows (BTA5:55370681), Tasmanian devils (SHA3:27733884, SHA3:27752896), tammar wallabies (MEUscaffold_38248:3028), and African clawed frogs (XLA9_10:68858749).

### 2.2. Evolutionary Conservation Analysis

**Figure 1:**
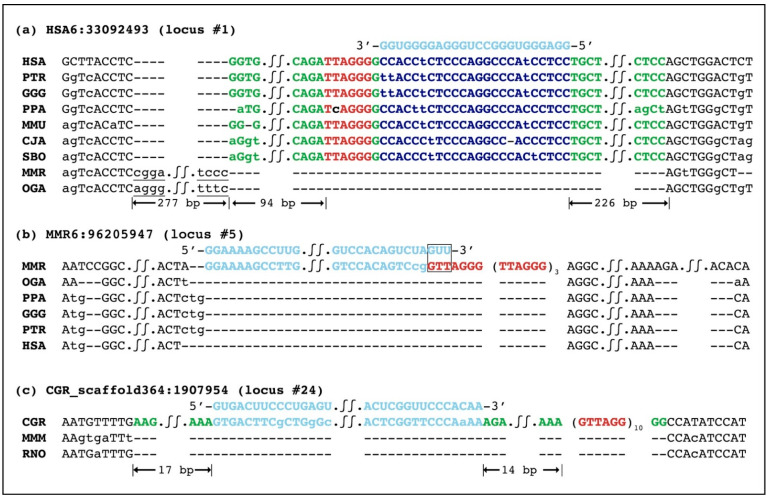
Examples of sequence alignments of orthologous TERC-ITS loci. For each locus, the top row shows the sequence of the telomerase RNA domain (light blue) homologous to the inserted TERC-like fragment. Species names are indicated on the left. Telomeric repeats are indicated in red. The TERC-like sequences homologous to a region of the telomerase RNA are indicated in light blue and the sequence complementary to a region of the telomerase RNA is in dark blue. Modifications of the insertion sites are shown: underlined nucleotides indicate deletions and green nucleotides indicate random sequence insertions. Nucleotides at the 3′ end of the TERC-like sequence in frame with the inserted telomeric repeats are boxed. (**a**) Simiiformes-specific TERC-ITS locus initially identified on human chromosome HSA6:33092493. The orthologous loci from mouse lemur and Northern galago are empty. (**b**) TERC-ITS at locus MMR6:96205947 from mouse lemur. The TERC-ITS is present only in the mouse lemur lineage, whereas in other primates the orthologous locus is empty. (**c**) TERC-ITS at locus CGR_scaffold364:1907954 specific to the Chinese hamster lineage. Orthologous loci from mouse and rat are empty.

**Figure 2:**
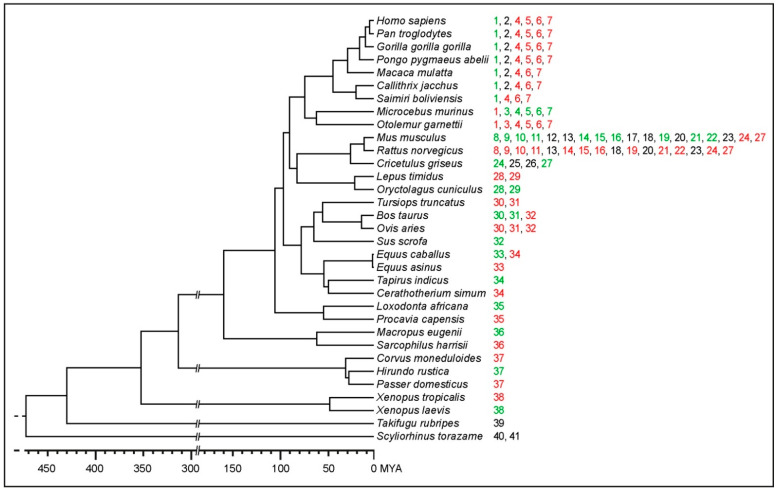
Phylogenetic tree of vertebrates showing species in which TERC-ITSs or empty orthologs were identified. For each species, loci are indicated on the right using the locus number reported in Table 1. Numbers in green indicate loci containing a TERC-ITS. Numbers in red indicate orthologous empty loci. Numbers in black indicate TERC-ITS loci for which no empty orthologs were identified. Where an orthologous locus was not found in a reference genome, its number is not reported.

For each TERC-ITS, we carried out a comparative sequence analysis with evolutionarily related genomes with the goal of analyzing their evolutionary conservation and, possibly, to identify the ancestral locus lacking the TERC-ITS insertion (Figure 1, Table 1, and Figures S1 and S2). To this purpose, for each locus, we downloaded a 1 kb sequence containing the TERC-ITS, 500 base pairs from the 3′ flanking region, and 500 base pairs from the 5′ flanking region. The sequence was used as the query for a BLAST search against the genome of evolutionarily related species. For a more precise identification of ancestral empty loci, the following additional species were included in the comparison: *Otolemur garnettii* (northern greater galago, OGA), *Lepus timidus* (mountain hare, LTI), *Ovis aries* (sheep, OAR), *Tursiops truncatus* (common bottlenose dolphin, TTR), *Ceratotherium simum simum* (Southern white rhinoceros, CSS), *Procavia capensis* (rock hyrax, PCA), *Passer domesticus* (house sparrow, PDO), *Corvus moneduloides* (New Caledonian crow, CMO), *Xenopus tropicalis* (Western clawed frog, XTR), *Danio rerio* (zebrafish, DRE), *Tetraodon nigroviridis* (green spotted puffer, TNI), *Oryzias latipes* (Japanese rice fish, OLA), *Chiloscyllium punctatum* (brownbanded bamboo shark, CPU), and *Rhincodon typus* (whale shark, RTY). The identification of empty loci allowed us to date TERC-ITS insertions during evolution (Figure 2) and to describe the DNA sequence modifications that occurred at the break sites (Figure 1, Figures S1 and S2, and Table S2).

In Figure 1, examples of sequence alignments of TERC-ITS loci with orthologous loci are shown, while in Figure 2, the identification number of each locus, as in Table 1, is reported near a phylogenetic tree of vertebrates.

For 29 out of the 41 TERC-ITS insertions, the identification of orthologous empty loci allowed us to date their insertion (Figure 2). The TERC-ITS shown in Figure 1a is conserved in HSA, PTR, GGG, PPA, MMU, CJA, and SBO, while an empty locus was found in MMR and OGA, indicating that this insertion occurred after the separation between Simiiformes primates and lemurs and before the separation between Old and New Word monkeys, that is, 42–75 million years ago. A 277 bp sequence (underlined in Figure 1a) that was present in the ancestral empty locus was deleted during the insertion event. The locus reported in Figure 1b is present only in MMR and therefore was inserted after the separation between the two lemur lineages analyzed here (Figure 2). In Figure 1c, a TERC-ITS locus that we identified in Chinese hamsters is shown. This insertion occurred in the CGR lineage after its separation from the mouse and rat lineage, that is, less than 30 million years ago (Figure 2). A 17 bp random sequence (green in Figure 1c) that is not present in the ancestral site was added at the break site during TERC-ITS insertion. In Figures S1 and S2, the comparative analysis of all TERC-ITS sequences identified in this work is shown. 

### 2.3. Insertion Site Characterization

The comparative analysis between TERC-ITSs and their ancestral empty loci showed that the insertions were usually accompanied by modifications of the break sites (Figure 1, Figures S1 and S2). In Table 2, the frequencies of the different types of modifications, including those previously observed by us in mice, rats [34], and horses [35], are reported. In Table S2, for each locus, the modification type detected is reported. Since for this analysis it was necessary to compare the sequence of TERC-ITSs with the one from orthologous empty loci, only 29 out of the 41 TERC-ITSs were informative. At five insertion sites, no modifications of the flanking sequences were observed. At 24 out of 29 informative loci, the introduction of TERC-ITSs occurred simultaneously with modifications of the insertion sites typical of the non-homologous end-joining repair pathway [36,41]: at 8 sites, the insertion occurred together with the deletion of a short sequence (underlined nucleotides in Figure 1, Figures S1 and S2); at 4 loci, the TERC-ITS was inserted together with an apparently random sequence (green nucleotides in Figure 1, Figures S1 and S2); at 12 loci, the TERC-ITS insertion was accompanied by a combination of deletion and random sequence addition. Similar to what we previously described at ITS loci, as well as at several TERC-ITSs, short microhomologies between the inserted sequence and the 3′ end of the break site were observed (yellow background in Figure S2).

At several loci, one to three nucleotides in frame with the inserted telomeric repeats were observed at the 3′ end of the TERC-like fragment (boxed nucleotides in Figure 1b, Figures S1d,e and S2d,f,g,k). For example, at the mouse lemur locus shown in Figure 1b, the region of the telomerase RNA homologous to the inserted TERC-like fragment terminates with a GUU trinucleotide (boxed) which is in frame with the inserted telomeric repeat stretch.

To further characterize the sequence features of the insertion sites, we analyzed the 2 kb regions upstream and downstream of each TERC-ITS, determining the GC and transposon content of each locus. The GC content varied greatly among the 41 loci, ranging between 30% and 64%, with an average value of 42.9%. Considering that the GC content of the vertebrate genomes analyzed here is comprised between 38.5%, in the cloudy catshark, and 45.7%, in fugu, we can conclude that the choice of the insertion site is not based on GC content. Using the UCSC table browser tool [42] (https://genome.ucsc.edu/cgi-bin/hgTables), we then evaluated the fraction of the 2 kb regions upstream and downstream of each TERC-ITS that was occupied by transposable elements (DNA transposons, LINESs, SINEs, and LTRs). Since good quality transposable element annotation is not available for all vertebrate species, this analysis was carried out on the 27 TERC-ITS loci found in Primates and Rodents (loci 1 to 27 in Table 1). Interestingly, at six loci, the TERC-ITS interrupted the consensus of a repetitive element: the TERC-ITS number 5 from mouse lemurs (Table 1 and Figure 1b) was inserted inside a SINE, the TERC-ITSs number 6 and 12 from mouse lemurs and mice, respectively (Table 1 and Figure S1d, [34]) were inserted inside LTRs, and the TERC-ITSs number 11 (Table 1 and [34]), number 21 (Table 1 and Figure S2b), and number 22 (Table 1 and [34]) from mice were inserted inside LINEs. As we previously showed [34], the telomeric repeat stretch at one TERC-ITS from rats (locus 13 in Table S2) was interrupted by a SINE. Comparative analysis showed that, whereas the TERC-ITS was conserved in both rats and mice, the SINE was rat-specific and was therefore inserted in the rat TERC-ITS after the separation between the mouse and the rat lineage. For the remaining 21 loci, the TERC-ITS was positioned at a distance comprised between 2 and 1664 nt from the closest transposon. The transposon content of sequences surrounding TERC-ITSs varied greatly among different loci, ranging between 14.9% and 83.4%, with an average value of 43%. According to RepeatMasker [43], in the vertebrate species analyzed here, the average fraction of genomic sequence occupied by mobile elements ranges between 38 and 48%. It is worth mentioning that these percentages can vary according to the number of transposons, but also according to the transposon annotation accuracy of each genome. Since the percentage of genomic DNA sequences which are occupied by transposons is very high, the presence of mobile elements within the analyzed 4 kb sequences is expected. Following a more detailed analysis of the fraction of genomic sequence at each locus occupied by the different classes of transposons (DNA transposons, LINESs, SINEs, and LTRs), we could not find enrichment for any specific class of mobile element. In conclusion, our observations suggest that there is no preferential insertion of TERC-ITSs based on transposon content.

### 2.4. Sequence Organization of TERC-ITS Loci

The length of the telomeric repeat array at TERC-ITS loci ranged from 6 to 205 bp and the length of TERC-like sequences ranged from 25 to 98 bp (Table S2). In the same Table, for each TERC-ITS, the position of the TERC-like fragment within each locus is reported together with its sequence conservation, as indicated by the number of mismatches compared to the corresponding *TERC* gene. 

**Figure 3:**
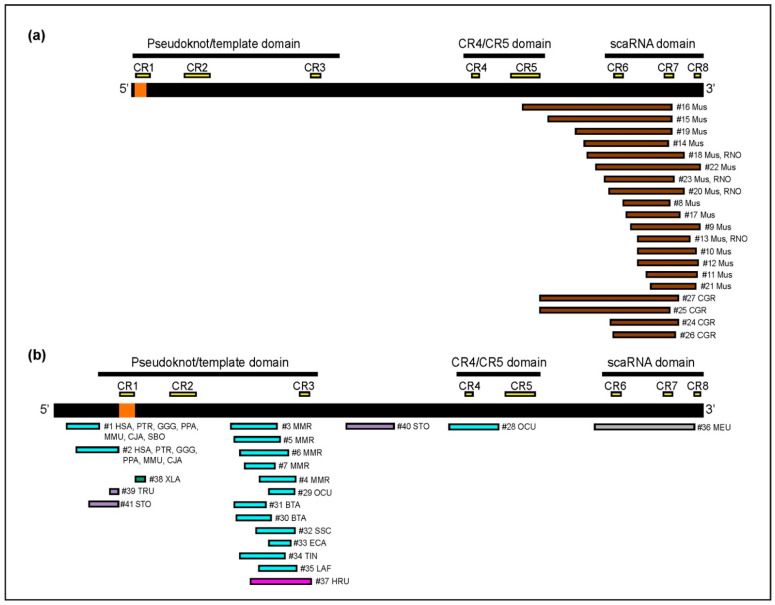
Sketch showing the portion of TERC included in each TERC-ITS. (**a**) The mouse TERC sequence is shown on top as a thick black line. Rodent TERC-like sequences are indicated as brown lines. (**b**) The human TERC sequence is shown on top. TERC-like sequences from non-rodent mammals are indicated as light blue lines, from marsupials as grey lines, from amphibians as dark green lines, from birds as pink lines, and from fish and shark as violet lines. On the TERC sequences, the position of the telomeric repeat template is marked in orange (CR1). The template region, which is retrotranscribed, by definition, at all loci, is not comprised in the colored lines.

In Figure 3, the portion of the TERC sequence included in each TERC-ITS locus is indicated. The 20 Rodentia TERC-like sequences are shown as straight lines under the mouse TERC, clearly showing that all of them are complementary to the scaRNA domain, close to the 3′ end of TERC, and in a few cases it also occupies a portion of the flanking region. On the contrary, the majority of the TERC-like sequences (18 out 21) from all the other species, in which the TERC RNA is characterized by a longer 5′ end, correspond to regions of the pseudoknot.

In Figure S3, the previously proposed secondary structure of the TERC RNA from eight species is sketched [6] and the overall regions retrotranscribed in TERC-ITSs are marked with a red line. Secondary structures of TERC RNAs were downloaded from the Telomerase Database (http://telomerase.asu.edu) [6,39,40]. Since, for some species, the secondary structure of the corresponding TERC was not available, the structure of the telomerase RNA from an evolutionarily related species was used (Table S2). 

**Figure 4:**
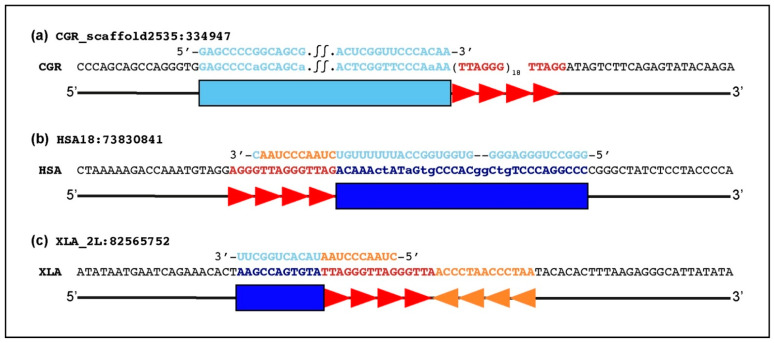
Organization of TERC-ITS loci. Sketches show the position and orientation of TERC-like sequences relative to TTAGGG repeats. Telomeric repeats in TTAGGG and CCCTAA orientation are indicated in red and orange, respectively. The TERC-like sequences homologous to a region of the telomerase RNA are indicated in light blue and the sequence complementary to a region of the telomerase RNA is in dark blue. (**a**) Organization of TERC-ITSs found in rodents, lemurs, lagomorphs, artiodactyls, perissodactyls, elephants, barn swallows, and cloudy catsharks (locus number 40). The Chinese hamster locus number 27 is shown as an example. The TERC-like sequence is upstream of the telomeric repeat array, and the two sequences are in an opposite orientation. Loci with this organization are marked with A in Table S2. (**b**) Organization of the TERC-ITSs found in Simiiformes primates, tiger pufferfish, and cloudy catsharks (locus number 41). Primate locus number 2 is shown as an example. The TERC-like sequence is downstream of the telomeric repeat array, and the two sequences are in the same orientation. Loci with this organization are marked with B in Table S2. (**c**) Organization of the TERC-ITS found in *Xenopus laevis*. The TERC-like sequence is positioned upstream of two stretches of telomeric hexamers organized head-to-head. This locus is marked with C in Table S2.

By analyzing the position and orientation of TERC-like sequences relative to TTAGGG hexamers, we could identify three types of TERC-ITS organizations (Figure 4). In 36 out of the 41 loci (A in Table S2), we observed the same organization previously described in mouse and horse loci (Figure 4a) [34,35]. At these loci, the TERC-like sequence is located upstream of the telomeric repeat and in the opposite orientation. In other words, telomeric repeats seem to have been directly retrotranscribed from the telomeric template at the 5′ domain of the telomerase RNA while the TERC-like region seems to be complementary to a retrotranscribed sequence. At four TERC-ITS loci (the two from Simiiformes primates, the one from fugu, and one of the two from the shark) (B in Table S2), the TERC-like sequence is positioned downstream of the telomeric repeat and in the same orientation (Figure 4b). Finally, at the locus from *Xenopus* (C in Table S2), the TERC-like sequence is positioned upstream of the telomeric repeat but in the same orientation (Figure 4c). In addition, at this complex locus, the ITS is composed of two telomeric repeat stretches in a head-to-head orientation.

## 3. Discussion

### 3.1. TERC-ITSs in Vertebrates

In 22 of the 30 vertebrate species analyzed here through BLAST search, we identified 41 TERC-ITS loci, demonstrating that TERC-ITSs are present throughout the vertebrate phylogenetic tree. 

It must be noted that, in our previous work [34], we were unable to find TERC-ITS loci in the human and chimpanzee genomes, while, in the present work, we identified two such loci that are present in Haplorrhini (*Homo sapiens*, *Pan troglodytes*, *Gorilla gorilla gorilla*, *Pongo pygmaeus abelii*, *Macaca mulatta*, *Callithrix jacchus*, and *Saimiri boliviensis*) while absent in Strepsirrhini (*Microcebus murinus* and *Otolemur garnettii*) and were therefore inserted in a common ancestor of Haplorrhini, that is, more than 42 million years ago (Table 1 and Table S2). This finding is a consequence of the less stringent search parameters adopted here. For one of these loci, it was possible to date the insertion event more precisely (42–75 MYA) because we identified the “empty” locus in lemurs (Figure 1 and Figure 2). In non-primate vertebrates, the identification of empty loci for 23 TERC-ITSs (Figure 2) allowed us to date their insertion. For the TERC-ITSs found in *Equus caballus, Mus musculus, Bos taurus*, and *Oryctolagus cuniculus*, the species containing the empty locus were relatively close and we could date their insertion at less than 3, 12, 17, and 22 MYA, respectively. For some loci, the evolutionary distance between the insertion containing and the insertion lacking species identified here did not allow a precise dating of the event. 

TERC-ITS loci have been found in the majority of the vertebrate species analyzed, but in eight species, no TERC-ITSs have been identified. This result may derive from poor quality of the genome assemblies and/or from the fact that some of these non-functional sequences may have accumulated mutations during evolution making them difficult to identify by BLAST. We previously demonstrated that, in the horse, some insertion sequences, such as ITSs and NUMTs (nuclear sequences of mitochondrial origin), are characterized by a particular type of polymorphism, that is, the presence of an insertion containing and an insertion missing (empty) allele in the population [35,44]. This insertion polymorphism characterizes sequences that have been introduced recently in the genome. For each species analyzed in the present study, we searched for TERC-ITSs in the reference genome and therefore it is possible that, at some polymorphic loci, only the empty allele was present in the analyzed individual. In line with all these considerations, we believe that the number of TERC-ITSs described here is underestimated. 

Interestingly, a particularly high number of TERC-ITSs is present in rodents, the mouse being the species containing the highest number (16 loci). In the mouse, TERC-ITSs are lineage specific and were therefore inserted recently during evolution. Their high number, may be related to the well described plasticity of the mouse genome, which contains a particularly large fraction of active transposons [45,46]. It is also possible that the high frequency of TERC-ITSs in rodents is due to the peculiar structure of their TERC, that is, the lack of 43 nucleotides at the 5′ end (Figure S3). The additional nucleotides at the 5′ end of non-rodent TERC may stabilize the pseudoknot/template domain [47] while, in rodent TERC, the missing nucleotides may allow greater flexibility of the 5′ and 3′ ends, favoring reverse transcription of the 3′ domain together with the telomeric repeat template.

Overall, the identification of TERC-ITSs throughout the vertebrate phylogenetic lineage indicates that they derive from evolutionarily conserved mechanisms.

TERC-like sequences derive mainly from three regions of TERC: (1) the scaRNA domain at the 3′ end, (2) sequences flanking the canonical telomeric repeat template in the pseudoknot/template at the 5′ end, and (3) the partially single-stranded region J2a/3 of the pseudoknot (Figure 3). Interestingly, at all rodent loci, the TERC-like portion derives from the 3′ end and, in a few cases, it extends to the adjacent region, while TERC-ITSs from non-rodent vertebrates contain insertions mainly deriving from the pseudoknot domain. There are only three exceptions: one of the two loci from *Scyliorhinus torazame* (locus 40 deriving from the sequence between Conserved Regions 3 and 4), one of the two loci from *Oryctolagus cuniculus* (locus 28 corresponding to Conserved Region 4 and its flanking sequence), and one from *Macropus eugenii* (locus 36). It should be mentioned that *Macropus eugenii*, whose TERC contains a relatively long 5′ domain similar to the human one, is the only non-rodent vertebrate among those analyzed that contains an insertion deriving from the scaRNA domain (Figure 3). Overall, these results suggest that the structure of TERC may influence the choice of the fragment to be retrotranscribed at TERC-ITS loci.

As sketched in Figure 4, the relative position and orientation of TERC-like sequences and telomeric repeats is variable. In the most frequent sequence organization (Figure 4a), that we observed in 36 out of the 41 loci (indicated as A in Table S2), the TERC-like sequence has been inserted upstream of the telomeric repeat and in the opposite orientation (Figure 4a). All rodent loci are characterized by this organization and, as mentioned above, the TERC-like fragment derives from the scaRNA domain and in a few cases also includes a portion of the adjacent region (Figure 3a). However, in the 16 non-rodent loci characterized by this sequence arrangement, the TERC-like fragment derives mainly from the partially single-stranded region J2a/3 of the pseudoknot (Figure 3b). At four loci (B in Supplementary Table S2), the TERC-like sequence is positioned downstream of the telomeric repeat and in the same orientation (Figure 4b). At these loci, the TERC-like fragment derives from the sequence immediately upstream of the telomeric repeat template within the telomerase RNA (Figure 3b). A third type of organization was observed at the *Xenopus* locus (C in Table S2): two head-to-head stretches of telomeric repeats are adjacent to a fragment deriving from a sequence immediately downstream of the telomeric repeat template (Figure 3b and Figure 4c).

In conclusion, although the number of loci analyzed so far is relatively small, in each evolutionary lineage, one kind of organization is prevalent, suggesting that lineage-specific features may influence the insertion mechanism.

### 3.2. Insertion Mechanisms of TERC-ITSs

Comparative genomic analysis allowed us to identify empty orthologous loci for the majority of TERC-ITSs. This analysis showed that TERC-ITSs appeared suddenly during evolution. Results from our previous studies indicate that ITSs and TERC-ITSs were inserted in genomes through a mechanism involving telomerase RNA as a template for the repair of DNA double-strand breaks [32,34,35]. The results presented here further support this hypothesis. In the present work, for the majority of the loci information for comparative analysis, the insertion of the TERC-ITS has been accompanied by modifications of the flanking sequences that we previously described for murine and equine TERC-ITSs [34,35] and for ITSs in primates [32], rodents [34], and horses [35], such as random nucleotide additions, deletions, and combinations of these two modifications. Our results are in agreement with previous observations from several authors, including us, showing that these sequence modifications typically occur during the repair of DNA double-strand breaks mediated by the non-homologous end-joining pathway [36,37,41,48,49]. As free ends arising from DNA double-strand breaks are often unsuitable for ligation, they are processed and made ligatable through these modifications. As a result, sites of repaired DNA double-strand breaks often display short deletions or sequence insertions like those observed at TERC-ITS loci. Altogether, these findings strongly suggest that TERC-ITSs were inserted during the repair of break sites in the course of evolution through reverse transcription of TERC sequences and the telomeric repeat array. It is worth mentioning that many DNA repair proteins also interact with telomeres and that, in turn, telomere binding factors can also be recruited at internal chromosomal loci and at DNA double-strand break sites [50,51,52,53,54]. In particular, it has been shown that Ku, a key player in the non-homologous end-joining DNA repair pathway with an affinity for double-strand ends, is also able to bind to the scaRNA domain of TERC [52,55]. It is reasonable that, on some occasions, TERC may be recruited at DNA double-strand breaks through interaction with Ku, and that TERT may be recruited as well thanks to its affinity for the telomerase RNA component. In addition, it is well known that telomerase is able to bind DNA breaks and perform so-called “chromosome healing”, creating new telomeric ends [56,57].

Is the choice of the insertion site random or driven by specific DNA sequence features? In agreement with our previous results [35], no relevant difference in terms of GC content between TERC-ITS loci and the average value of the genome assembly has been detected, indicating that the choice of the insertion site was not preferentially based on this characteristic. Similarly, although 6 of the 27 TERC-ITSs found in primates and rodents were inserted within transposable elements, the analysis of transposon density in sequences flanking TERC-ITSs showed no relevant enrichment compared to the average value of the genome. This observation suggests that transposons were not involved in the dissemination of TERC-ITSs in genomes.

**Figure 5:**
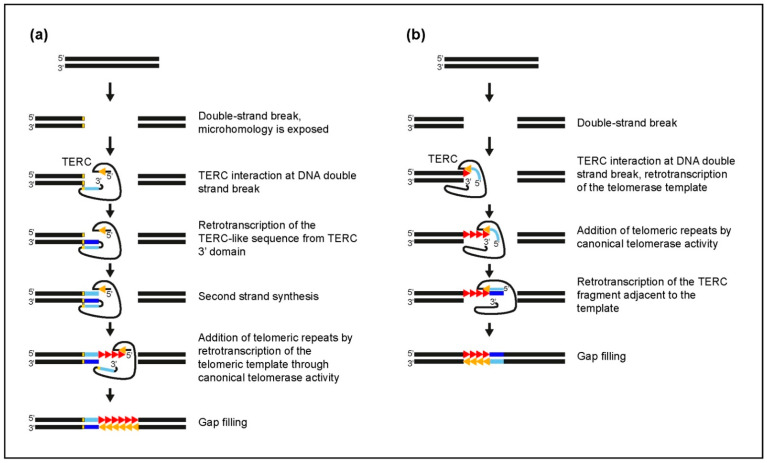
Models explaining the insertion of TERC-ITSs at break sites. (**a**) Insertion of a TERC-ITS containing a TERC-like fragment and a telomeric repeat array in an opposite orientation. This model (modified from [34]) describes the origin of TERC-ITSs containing sequences homologous to the 3′ end of TERC. After a double-strand break, TERC interacts with one side of the break exposing its 3′ domain. Microhomologies (yellow lines) may favor the interaction. The 3′ end of telomerase RNA may then fold back, acting as a primer for retrotranscription of the scaRNA domain (the dark blue line represents the sequence retrotranscribed from the 3′ domain of TERC). The sequence retrotranscribed from the 3′ domain of TERC is used as a template to synthesize the second DNA strand (light blue line). This step is followed by retrotranscription of TTAGGG repeats (red triangles) from the telomeric repeat template (orange triangle) through the canonical telomerase enzyme activity. (**b**) Mechanism proposed for the insertion of loci in which the TERC fragment and the telomeric repeats are in the same orientation. Telomeric repeats are retrotranscribed by telomerase using a 3′ end of the DNA break site as a primer. The addition of the last telomeric repeat is followed by the retrotranscription of an adjacent portion of the TERC RNA.

Since in rodent TERC-ITSs the TERC fragment and the telomeric repeats are in an opposite orientation to each other, we previously proposed a complex model to explain their insertion at break sites [21,34]. This model can be extended to the non-rodent loci characterized by a similar organization. A sketch of this type of loci is shown in Figure 4a and the loci are listed in Table S2, marked with an A. In Figure 5a. the model that was described in detail in our previous work [34] is re-proposed. We suggested that, following a DNA double-strand break, the 3′ end of TERC may interact with one of the extremities of the break site. Microhomologies between one end of the double-strand break and the 3′ end of TERC may help to stabilize this interaction. The 3′ end of telomerase RNA may then fold back, providing a free 3′OH acting as a primer for retrotranscription of the scaRNA domain. The addition of telomeric repeats may be facilitated by pairing of the template region of TERC with short microhomologies with the 3′ end of the inserted TERC fragment (boxed nucleotides in Figure 1, Figures S1 and S2). Synthesis of the second strand, addition of telomeric repeats, and gap filling should complete the TERC-ITS insertion. This model can explain the peculiarities of orientation and sequence composition of the inserts containing fragments deriving from the scaRNA domain (loci 8–27, 36 in Table S2 and Figure 3). 

To explain the insertion of TERC-like fragments far away from the 5′ end and different from the scaRNA domain (loci 3–7, 28–35, 37, 40 in Table S2 and Figure 3), we now propose that short versions of TERC may participate in the process and be retrotranscribed, leading to the insertion of the J2a/3 or the CR4/CR5 domains. The recent description of a short form of TERC which can interact with telomerase and participate in its regulation [58] supports this model. At two mouse lemur loci (Figure S1d,e), two head-to-head stretches of telomeric repeats were observed. Since it was demonstrated that telomerase can function as a dimer in vivo [59], we propose that a second enzyme may have added telomeric repeats to the other side of the DNA break. 

For the loci in which the TERC fragment and the telomeric repeats are in the same orientation, as shown in Figure 4b (1, 2, 39, 41 in Table S2 and Figure 3), a simpler mechanism can be proposed for their insertion (Figure 5b): the telomeric repeats are retrotranscribed by telomerase using a 3′ end of the DNA break site as a primer. The addition of the last telomeric repeat is followed by retrotranscription of an adjacent portion of the TERC RNA by telomerase or by another reverse transcriptase. The organization of the *Xenopus laevis* locus, comprising a head-to-head stretch of telomeric repeats (Figure 4c), should involve retrotranscription of the TERC fragment immediately adjacent to the template followed by the addition of telomeric repeats. As for the mouse lemur loci mentioned above, the intervention of a second telomerase enzyme can be proposed for the insertion of telomeric repeats at the other side of the DNA break. Overall, at 3 TERC-ITSs out of 41, we observed this organization of the telomeric repeat, suggesting that the proposed intervention of a second enzyme may occur rarely during the insertion of TERC-ITSs.

It has been shown that extrachromosomal circular molecules of DNA composed by telomeric repeats (t-circles) can contribute to telomere maintenance though homologous recombination-mediated mechanisms [60]. Is it possible that TERC-ITSs are generated through such a mechanism involving t-circles? Although this hypothesis is fascinating, it is unlikely that such mechanisms are responsible for the insertion of TERC-ITSs in genomes. Firstly, our data show that no homologous sequences allowing recombination between empty loci and t-circles are present in the ancestral empty loci. Moreover, the simultaneous insertion of telomeric repeats and TERC-like fragments during the generation of TERC-ITSs would imply that t-circles and TERC are recruited at break sites at the same time and used as substrate for the generation of TERC-like sequences and telomeric repeat arrays. It is unlikely that TERC-ITSs derive from such a complicated mechanism that would involve different substrates and enzymatic machinery.

As a final consideration, the results presented here demonstrate that TERC-ITSs were inserted in the genome of a wide range of vertebrate species through a mechanism involving telomerase RNA as a template for the repair of DNA double-strand breaks. We do not know how often and in which cell types a break can be repaired through the mechanisms described here. A TERC-ITS, to be detected in the genome of a species, must have been inserted during the repair of a break that occurred in the germline and was fixed in the population. However, such mechanisms are probably operating in all vertebrates, adding a novel biological function to the non-canonical roles of telomerase.

## 4. Materials and Methods

### 4.1. Identification of TERC Genes in 30 Vertebrate Species

Thirty vertebrate species were selected from different orders on the basis of the availability of an identifiable *TERC* gene in the Telomerase Database (http://telomerase.asu.edu) [39,40] and of a well assembled genomic sequence from NCBI (https://www.ncbi.nlm.nih.gov/assembly) [61]. Analyzed genomes were from HSA (GRCh38.p13, March 2019), PTR (Clint_PTRv2, January 2018), GGG (Kamilah_GGO_v0, August 2019), PPA (Susie_PABv2, January 2018), MMU (Mmul_10, February 2019), CJA (Callithrix_jacchus_cj1700_1.1, May 2020), SBO (saiBol1, November 2011), MMR (Mmur_3.0, February 2017), Mus (GRCm39, July 2020), RNO (mRatBN7.2, November 2020), CGR (CriGri_1.0, August 2011), OCU (OryCun2.0, October 2009), FCA (Felis_catus_9.0, February 2017), CLF (ROS_Cfam_1.0, September 2020), BTA (ARS-UCD1.2, April 2018), SSC (Sscrofa11.1, February 2017), ECA (EquCab3.0, January 2018), TIN (TapInd_v1_BIUU, January 2019), LAF (LoxAfr3.0, July 2009), SHA (mSarHar1.11, November 2019), MEU (Meug_1.1, November 2009), HRU (bHirRus1.pri.v2, March 2021), GGA (GRCg6a, April 2018), AHY (ASM993644v1, January 2020), XLA (Xenopus_laevis_v2, August 2016), TRU (FTakRub1.2, June 2019), STO (Storazame_v1.0, August 2018), PMA (kPetMar1.pri, February 2020), LCA (LetJap1.0, September 2013), and EBU (Eburgeri_3.2, October 2017).

For 17 species (HSA, MMU, CJA, Mus, RNO, OCU, ECA, BTA, SSC, FCA, GGA, AHY, XLA, TRU, PMA, LCA, and EBU), the *TERC* gene and the corresponding RNA sequence were available in the Telomerase Database (http://telomerase.asu.edu) [39,40]. For the remaining 13 species (PTR, GGG, PPA, SBO, MMR, CGR, CLF, TIN, LAF, SHA, MEU, HRU, and STO), the TERC sequence was not annotated. For these species, the putative *TERC* gene was BLAST-searched (https://blast.ncbi.nlm.nih.gov/Blast.cgi) [62] against the corresponding genome using the sequence of evolutionarily related species as the query. The search was performed using this setup: blastn algorithm, max target sequences = 100; automatically adjust parameters for short input sequences = disabled; expect threshold = 10; word size = 11; max matches in a query range = 0; match/mismatch scores = 2, –3; gap costs = existence:5, extension:2, and filters for low complexity regions and species-specific repeats disabled. The automatic adjustment of search parameters for short sequences was disabled. Table S1 contains the complete list of the TERC sequences, the version of the genome assembly used for the analysis, the coordinates of each sequence in the assembly, and the NCBI accession number of the gene. For those species whose *TERC* gene was not annotated, the name of the species whose TERC was used as the query to BLAST-search the putative gene is indicated.

### 4.2. Search for TERC-ITS Loci in Vertebrate Genomes

TERC-like sequences were BLAST-searched in the genomic assembly of each species using the sequence of the corresponding *TERC* gene as the query (https://blast.ncbi.nlm.nih.gov/Blast.cgi) [62]. BLAST was set up as described above. To identify TERC-ITSs, we scanned the sequences adjacent to each BLAST hit for the presence of telomeric hexamers. For this search, loci comprising at least one telomeric repeat were considered. In total, we identified 41 TERC-ITSs in 22 out of 30 species.

To assess the evolutionary conservation of each TERC-ITS, for each locus we downloaded a 1 kb sequence containing the TERC-ITS: 500 base pairs from the 3′ flanking region and 500 base pairs from the 5′ flanking region. Sequences were used as the query for a BLAST search against the genome of evolutionarily related species. For a more precise evolutionary analysis of the loci, the following additional species were also analyzed: OGA (OtoGar3, March 2011), LTI (CIBIO-ISEM_LeTim_1.1, July 2020), OAR (Oar_rambouillet_v1.0, October 2017), TTR (mTurTru1.mat.Y, March 2020), CSS (CerSimSim1.0, July 2012), PCA (ProCapCap_v2_BIUU_UCD, July 2019), PDO (Passer_domesticus-1.0, August 2016), CMO (UO_Cmon_1.0, November 2019), XTR (UCB_Xtro_10.0, November 2019), DRE (GRCz11, May 2017), TNI (ASM18073v1, May 2004), OLA (ASM223467v1, July 2017), CPU (Cpunctatum_v1.0, August 2018), and RTY (ASM164234v2, March 2017). BLAST was set up as previously described. Sequences were aligned using the multiple sequence alignment software, MultAlin (http://multalin.toulouse.inra.fr/multalin) [63,64]. The UCSC Table Browser tool (https://genome.ucsc.edu/cgi-bin/hgTables) [42] and the RepeatMasker software (http://www.repeatmasker.org) [43] were utilized to identify known repetitive sequences.

TimeTree (http://www.timetree.org) [65,66] was used to build the phylogenetic tree of vertebrate species containing TERC-ITSs or empty orthologous loci. When required, the tree was modified to include species or to adjust the date of the divergence of two or more species based on the literature.

## Figures and Tables

**Table 1:** Vertebrate TERC-ITS loci.

Number	Species	Chromosomal Localization	Starting Nucleotide of the TERC-Like Sequence (Length)	Position within the TERC Sequence	ITS Length (bp)	Orthologous TERC-ITS Loci	Orthologous Empty Loci
1	*Homo sapiens*	HSA6	33092493 (25)	8–32	6	PTR, GGG, PPA, MMU, CJA, SBO	MMR, OGA
2	*Homo sapiens*	HSA18	73830841 (31)	15–45	14	PTR, GGG, PPA, MMU, CJA	ND
3	*Microcebus murinus*	MMR3	9808477 (35)	120–154	43	ND	OGA
4	*Microcebus murinus*	MMR4	11270478 (27)	142–168	43	ND	HSA, PTR, GGG, PPA, MMU, CJA, SBO, OGA
5	*Microcebus murinus*	MMR6	96205947 (34)	123–157	24	ND	HSA, PTR, GGG, PPA, OGA
6	*Microcebus murinus*	MMR14	66686502 (36)	127–162	32	ND	HSA, PTR, GGG, PPA, MMU, CJA, SBO, OGA
7	*Microcebus murinus*	MMR32	2429016 (28)	129–156	38	ND	HSA, PTR, GGG, PPA, MMU, CJA, SBO, OGA
8	*Mus musculus* ^1^	Mus1	46771696 (31)	341–374	57	ND	RNO
9	*Mus musculus* ^1^	Mus1	69071165 (50)	346–395	139	ND	RNO
10	*Mus musculus* ^1^	Mus4	119841389 (42)	351–392	53	ND	RNO
11	*Mus musculus* ^1^	Mus5	25644087 (37)	357–393	21	ND	RNO
12	*Mus musculus* ^1^	Mus6	67988979 (44)	351–394	93	ND	ND
13	*Mus musculus* ^1^	Mus8	23257793 (38)	351–388	213	RNO	ND
14	*Mus musculus* ^1^	Mus9	47869714 (60)	314–373	68	ND	RNO
15	*Mus musculus* ^1^	Mus10	20335691 (98)	289–388	108	ND	RNO
16	*Mus musculus* ^1^	Mus10	58382382 (118)	271–388	27	ND	RNO
17	*Mus musculus* ^1^	Mus11	86905013 (38)	343–381	55	ND	ND
18	*Mus musculus*	Mus12	7976282 (68)	316–384	77	RNO	ND
19	*Mus musculus* ^1^	Mus12	111293353 (81)	308–388	13	ND	RNO
20	*Mus musculus* ^1^	Mus13	3641558 (54)	331–384	22	RNO	ND
21	*Mus musculus*	Mus17	77320142 (33)	360–392	58	ND	RNO
22	*Mus musculus* ^1^	MusX	65734790 (74)	322–395	23	ND	RNO
23	*Mus musculus* ^1^	MusX	99124249 (52)	328–377	25	RNO	ND
24	*Cricetulus griseus*	CGR_scaffold364	1907954 (49)	332–380	60	ND	Mus, RNO
25	*Cricetulus griseus*	CGR_scaffold477	2816499 (92)	283–374	205	ND	ND
26	*Cricetulus griseus*	CGR_scaffold628	177514 (44)	336–379	35	ND	ND
27	*Cricetulus griseus*	CGR_scaffold2535	334947 (98)	283–380	113	ND	Mus, RNO
28	*Oryctolagus cuniculus*	OCU20	24654676 (37)	245–281	66	ND	LTI
29	*Oryctolagus cuniculus*	OCU_chrUn0052	1868849 (20)	148–167	39	ND	LTI
30	*Bos taurus*	BTA12	22246021 (26)	125–150	24	ND	OAR, SSC, TTR
31	*Bos taurus*	BTA20	7319073 (24)	124–147	23	ND	OAR, SSC, TTR
32	*Sus scrofa*	SSC7	70772067 (29)	139–167	40	ND	BTA, OAR
33	*Equus caballus* ^1^	ECA19	10034300 (17)	148–164	35	ND	EAS
34	*Tapirus indicus*	TIN_scaffold2309	127238 (33)	128–160	26	ND	CSS, ECA
35	*Loxodonta africana*	LAF_scaffold21	34372782 (28)	141–168	19	ND	PCA
36	*Macropus eugenii*	MEU_scaffold107834	53871 (79)	424–502	12	ND	SHA
37	*Hirundo rustica*	HRU5	20102963 (42)	106–146	10	ND	PDO, CMO
38	*Xenopus laevis*	XLA_2L	82565752 (11)	54–64	28	ND	XTR
39	*Takifugu rubripes*	TRU18	10170798 (8)	7–14	46	ND	ND
40	*Scyliorhinus torazame*	STO_scyto00000011	2181744 (31)	233–263	30	ND	ND
41	*Scyliorhinus torazame*	STO_scf_scyto00006538	68375 (16)	4–19	27	ND	ND

^1^ Loci previously identified [34,35].

**Table 2:** Flanking sequence modifications at TERC-ITS loci.

Flanking Sequence Modification	Number of Loci (%)
No modification	5 (17.2)
Deletion	8 (27.6)
Insertion	4 (13.8)
Deletion and Insertion	12 (41.4)
TOTAL	29 (100)

## Data Availability

No new data were created or analyzed in this study. Sequences were retrieved from NCBI (https://www.ncbi.nlm.nih.gov/assembly) and Telomerase Database (http://telomerase.asu.edu).

## Supplementary Materials

Supplementary materials can be found at https://www.mdpi.com/article/10.3390/ijms222011048/s1.
