# Supplementary material for: Telomeric-Like Repeats Flanked by Sequences Retrotranscribed from the Telomerase RNA Inserted at DNA Double-Strand Break Sites during Vertebrate Genome Evolution
Source: Int J Mol Sci. 2021 Oct 13;22(20):11048. doi: 10.3390/ijms222011048 (PMC8537989; doi:10.3390/ijms222011048)
Supplement: Supplementary file 1 [file ijms-22-11048-s001.zip › ijms-1395546-supplementary.pdf]

Table S1. Sequences of TERC genes used to BLAST-Search TERC-ITS loci. The sequence corresponding to the telomeric repeat template is in bold.

Species	Assembly	TERC sequence from genomic assembly used to BLAST-search TERC-ITSs	Coordinates	TERC NCBI accession number (when available)	Species used to identify the TERC sequences with BLAST
Homo sapiens	GRCh38.p13<br>(28/02/2019)	1 GGGTTGCGGA GGGTGGGCCT GGGAGGGGTG GTGGCCATTT TTTGT**CTAAC CCTAA**CTGAG AAGGGCGTAG<br>71 GCGCCGTGCT TTTGCTCCCC GCGCGCTGTT TTTCTCGCTG ACTTTCAGCG GGCGGAAAAG CCTCGGCCTG<br>141 CCGCCTTCCA CCGTTCATTC TAGAGCAAAC AAAAAATGTC AGCTGCTGGC CCGTTCGCCC CTCCCGGGGA<br>211 CCTGCGGCGG GTCGCCTGCC CAGCCCCCGA ACCCCGCCTG GAGGCCGCGG TCGGCCCGGG GCTTCTCCGG<br>281 AGGCACCCAC TGCCACCGCG AAGAGTTGGG CTCTGTCAGC CGCGGGTCTC TCGGGGGCGA GGGCGAGGTT<br>351 CAGGCCTTTC AGGCCGCAGG AAGAGGAACG GAGCGAGTCC CCGCGCGCGG CGCGATTCCC TGAGCTGTGG<br>421 GACGTGCACC CAGGACTCGG CTCACACATG C	chr3:169764610-169765060	NR_001566	*Homo sapiens*<br>(NR_001566)
Pan troglodytes	Clint_PTRv2<br>(19/01/2018)	1 GGGTTGCGGA GGGTGGGCCT GGGAGGGGTG GTGGCCATTT TTTGT**CTAAC CCTAA**CTGAG AAGGGCGTAG<br>71 GCGCCGTGCT TTTGCTCCCC GCGCGCTGTT TTTCTCGCTG ACTTTCAGCG GGCGGAAAAG CCTCGGCCTG<br>141 CCGCCTTCCA CCGTTCATTC TGGAGCAAAC AAAAAATGTC AGCTGCTGGC CCGTTCGCCC CTCCCGGGGA<br>211 CCTGCGGCGG GTCGCCTGCC CAGCCCCCGA ACCCCGCCTG GAGGCCGCGG TCGGCCCGGG GCTTCTCCGG<br>281 AGGCACCCAC TGCCGCCGCG AAGAGTTGGG CTCTGTCAGC CGCGGGTCTC TCGGGGGCGA GGGCGAGGTT<br>351 CAGGCCTTTC AGGCCGCAGG AAGAGGAACG GAGCGAGTCC CCGCGCGCGG CGCGATTCCC TGAGCTGTGG<br>421 GACGTGCACC CAGGACTCGG CTCACACATG	chr3:167800495-167800944	No	*Homo sapiens*<br>(NR_001566)
Gorilla gorilla gorilla	Kamilah_GGO_v0<br>(28/08/2019)	1 GGGTTGCGGA GGGTGGGCCT GGGAGGGGTG GTGGCCATTT TTTGT**CTAAC CCTAA**CTGAG AAGGGCGTAG<br>71 GCGCCGTGCT TTTGCTCCCC GCGCGCTGTT TTTCTCGCTG ACTTTCAGCG GGCGGAAAAG CCTCGGCCTG<br>141 CCGCCTTCCA CCGTTCATTC TGGAGCAAAC AAAAAATGTC AGCTGCTGGC CCGTTCGCCC CTCCCGGGGA<br>211 CCTGCGGCGG GTCGCCTGCC CAGCCCCCGA ACCCCGCCTG GAGGCCGCGG TCGGCCCGGG GCTTCTCCGG<br>281 AGGCACCCAC TGCCGCCGCG AAGAGTTGGG CTCTGTCAGC CGCGGGTCTC TCGGGGGCGA GGGCGAGGTT<br>351 CAGGCCTTTC AGGCCGCAGG AAGAGGAACG GAGCGAGTCC CCGCGCGCGG CGCGCTTCCC TGAGCTGTGG<br>421 GACGTGCACC CAGGACTCGG CTCACACATG C	chr3:166181647-166182097	No	*Homo sapiens*<br>(NR_001566)
Pongo pygmaeus abelii	Susie_PABv2<br>(19/01/2018)	1 GGGTTGCGGA GGGTGGGCCT GGGAGGGGTG GTGGCCATTT TTTGT**CTAAC CCTAA**CTGAG AAGGGCGGTC<br>71 GCGCCGTGCT TTTGCTCCCC GCGCGCTGTT TTTTTCGCTG ACTTTCAGCG GGCGGAAAAG CCTCGGCCTG<br>141 CCGCCTTCCA CCGTTCATTC TGGAGCAAAC AAAAAATGTC AGCTGCTGGC CCGTTCGCCC CTCCCGGGGA<br>211 CCTGCGGCGG GTCGCCTGCC CAGCCCCCGA ACCCCGCCTG GAGGCCGCGG TCGGCCCGGG GCTTCTCCGG<br>281 AGGCACCCAC TGCCGCCGCG AAGAGTTGGG CTCTGTCAGC CGCGGGTCTC TCGGGGGCGA GGGCGAGGTT<br>351 CAGGCCTTTC AGGCCGCAGG AAGAGGAACG GAGCGAGTTC CCGCGCGCGG CGCGCTTCCC TGAGCTGTGG<br>421 GACGTGCACC CAGGACTCGG CTCACACT GC	chr3:167351202-167351648	No	*Homo sapiens*<br>(NR_001566)
Macaca mulatta	Mmul_10<br>(13/02/2019)	1 GGGTTGCGGA GGGTGGGCCT GGGAGAGGTG GCGGCCATTT TTTGT**CTAAC CCTAA**CTGAG AAGGGCGTAG<br>71 GCGCCGCGCT TTTGCTCCCC GCGCGCTGTT TTTCTCGCTG ACTTTCAGCG GGCGGAAAAG CCTCGGCCTA<br>141 CCGCCTTCCA CCGTTCATTC TGGAGCAAAC AAAAAATGTC AGCTGCTGGC CCGTTCGCCC CTCCCGGGGA<br>211 CCTGCGGCGG GTCGCCTGCC CAGCCCCCGA ACCCCGCCTG GAGGCCGCGG TCGGCCCGGG GCTTCTCCGG<br>281 AGGCGCCCAT TGTCGCCGCG AAGAGTTGGG CTCTGTCAGC CGCGGGTCTC TCGGGGGTGA GGGCGAGGTA<br>351 CAGGCCTTTC AGGCCGCAGG AAGAGGAACG GAGCGGAGTC CCCGCGCGCG GCGCGCTTCC CTGAGCTGTG<br>421 GGACGTGCAC CCAGGACTCG GCTCACACAT GCAG	chr2:74696847-74697300	NR_033816	*Macaca mulatta*<br>(NR_033816)
Callithrix jacchus	Callithrix_jacchus_c<br>j1700_1.1<br>(22/05/2020)	1 GGGTTGCGGA GGGTGGGCCT GGGAGGGTGG TGGCCATTTT TTTT**CTAACC CTAA**CTGAGA CGGGCGTAGG<br>71 CGCCGTGCTT TTGCTCCCCG CGCGCTGTTT TTCTCGCTGA CTTTCAGCGT TCGGAAAAGC CTCGGCCTAC<br>141 CGCCTTCCAC CGTTCATTCT GGAGCAAACA AAAAATGTCA GCCGCTGGCC AGTTCGCCCC TCCCGGGGAC<br>211 CTGCGGCGGG TAGCCTGCCT AGCCCCCGAA CCCCGCCTGG AGGCCGCGGT CGGCCCGGGG CTTCTCCGGA<br>281 GGCGCCCATT GCCGCCGCGA AGAGTTGGGC TCTGTCAGCC GCGGGTGTCT CGGGGGCGAG GGTGAGGCTC<br>351 AGGCCTTTCA GGCCGCAGGA AGAGGAACGG AGCGAGTTCC CACGCACGGC GCGCTTCCCT GAGCTGTGGG<br>421 ACGTGCACCC AGAACTCGGC TCACACATGC AGT	chr17:10422651-10423104	NR_033817	*Callithrix jacchus*<br>(NR_033817)

Saimiri boliviensis	saiBol1 (17/11/2011)	1 GGGTTGCGGA GGGTGGGCCT GGGAGGGGTG GCGGCCATTT TTTCT**CTAAC CCTAA**CTGAG ACGGGCGTAG<br>71 GCGCCGTGCT TTTGCTCCCC GCGCGCTGTT TTTCTCGCTG ACTTTCAGCG TTCGGAAAAG CCTCGGCCTA<br>141 CCGCCTTCTA CCGTTCATTC TGGAGCAAAC AAAAAATGTC AGCCGCTGGC CTGTTCGCCC CTCCCGGGGA<br>211 CCTGCGGCGG GTAGCCTGCC CAGTCCCCGA ACCCCGCCTG GAGGCCGCGG TCGGCCCGGG GCTTCTCCGG<br>281 AGGCGCCCAT TGCCGCCGCG AAGAGTTCGG CTCTGTCAGC CGCGGGTGTC TCGGGGGCGA GGGTGAGGCT<br>351 CAGGCCTTTC AGGCCGCAGG AAGAGGAACG GAGCGAGTTC CCGCGCGCGG CGCGCTTCCC TGAGCTGTGG<br>421 GACGTGCACC CAGAACTCGG CTCACACATG CAGT	Scaffold00013:8978910-8979363	No	*Callithrix jaccus* (NR_033817)
Microcebus murinus	Mmur_3.0 (06/02/2017)	1 GGGTTGCGGA GGGTGGGCCC GGGAGGGGTG TCGGCCATTA GTGC**CTAACC CTAA**CTGAGA AGGGCGTAGG<br>71 CGCCGTGCTT TTGCTTCCCG CGCGCTGTTT TTCTCGCTGA CTTTCAGCGG GCGGAAAAGC CTTGGCCTAC<br>141 TGCCGTCCAC AGTCTAGTTT GGAGCAAACA AAAAATGTCA GCGCTGGCCG GTTCACCCCT CCCGGGACCT<br>211 GCGGTGGCTC GCCCGCCCTG CCCCGAGCCC TGCCTAAAGG CCGCGGTCGA CCTGGGGCTT CTCCGGAGGT<br>281 GCCCAATGTC GCCGCGAAGA GTTGGGCTCT GTCAGCCGCG GGTCCTTCGG GTGCCAAGGG CGAGGCTCAG<br>351 GCCTGCTGGC CGCAGGAAAG TAACGGAGCG GGTCCCTTCA CGCGGTGCGC TTCCCTGAGC AGTGGGACTT<br>421 GCGCCCGGGA CTCAGCTCCT ACATACATC	chr1:63529673-63530121	No	*Callithrix jaccus* (NR_033817)
*Mus musculus**	GRCm39/mm39 (07/2020)	1 AC**CTAACCCT** GATTTTCATT AGCTGTGGGT TCTGGTCTTT TGTTCTCCGC CCGCTGTTTT TCTCGCTGAC<br>71 TTCCAGCGGG CCAGGAAAGT CCAGACCTGC AGCGGGCCAC CGCGCGTTCC CGAGCCTCAA AAACAAACGT<br>141 CAGCGCAGGA GCTCCAGGTT CGCCGGGAGC TCCGCGGCGC CGGGCCGCCC AGTCCCGTAC CCGCCTACAG<br>211 GCCGCGGCCG GCCTGGGGTC TTAGGACTCC GCTGCCGCCG CGAAGAGCTC GCCTCTGTCA GCCGCGGGGC<br>281 GCCGGGGGCT GGGGCCAGGC CGGGCGAGCG CCGCGAGGAC AGGAATGGAA CTGGTCCCCG TGTTCGGTGT<br>351 CTTACCTGAG CTGTGGGAAG TGCACCCGGA ACTCGGTTCT CACAACC	chr3:96321753-96322149	NR_001579.1	*Mus musculus* (NR_001579)
*Rattus norvegicus**	mRatBN7.2 (10/11/2020)	1 GT**CTAACCCT** ATTGTTATAG CTGTGGGTTC TGTTCTTTTG TTCTCCGCCC GCTGTTTTTC TCGCTGACTT<br>71 TCAGCGGGCC TGGAAAGTTC AGACCTGCAG CGGGTCACCG CGCATTCTGG AACCTCAAAA AATGTCAGCG<br>141 TAGGAGCTCT GGTGCCAGAG CTCCGCGGCG CTGGGCCGCC CAGCCCGGTA CCCGCCTGGA GGCCGCGGAC<br>211 GGCCTGGGGT CTTAGAACTC CGCTGCCGCC GTGAAGAGCT AGTCTCTGTT AGCTACGGGG CACCGGGCGC<br>281 TGGGGTCAGG CCGGGAGAGC GCCGCAAGGA CAGTAACGGA ACTGGTCCCT GAGTTCGGTG GCTTTCCTGA<br>351 GATGTGGGAA GTGCACCTGG AACTCAGTTC CTACAACC	chr2:112815654-112816041	NR_001567.1	*Rattus norvegicus* (NR_001567)
Cricetulus griseus	CriGri_1.0 (23/08/2011)	1 GT**CTAACCCT** GAATTCTGAG AGCTGTGGGT ACTGTGCTTT CGTCTCCGCC CGCTGTTTTT CTCGCTGACT<br>71 TCCAGCGGGC GGGAAAGTCC AGACCTGCAG CGGGCCATCG CGCGTTTTCC ACCACAAAAA AATGTCAGCG<br>141 CTGGGCTCAT GTGCCTGGAG CCTTGCGGCC GGCCGCCCAG CCCCGCACCC GCCTGAGGCC GCGGTCGGCC<br>211 TGGAGTCCTC GGGCTCCGCT GCCGCCGCGA AGAGCTAGAC TCTGTCAGCC GCGGGGCGTC AGGGGCTGGG<br>281 GCGAGCCCCG GCAGCGCCGC AAGCAGAGAA ACGGAGCTGG TCCCGTGAAC GGTGACTTCC CTGAGTTGTG<br>351 GGAAATGCAC CAGGAACTCG GTTCCCACAA CC	scaffold2001:731353-731734	No	*Mus musculus* (NR_001579)
Oryctolagus cuniculus	OryCun2.0 (20/10/2009)	1 GGGCTGAGGA GGGTGGGCTC GGGAGGGGCC CGGTCATTTC TCAT**CTAACC CTAA**CTGAGC AGGGCGTAGG<br>71 CGCCGCGCTT TTGTTTCCCC GCGCGCTGTT TTTCTCGCTG ACTTTCAGCG CGTGGGAAAA GCCTTGGCCT<br>141 ACCGCCGTCC ACCGTTCATT TCGCAGTAAA CAAAAAATGT CAGCCGCTGG CCGGTTCGCC CTTCCCGGGG<br>211 ACCTGCGGTG GCTCGCCCGC CCGGCCCCCG TGCCCCGCCT GAGGCCGCGG TCGGCCCGGG GCTTCTCCGG<br>281 AGGTGCCCAA TGCCGCCGCG AAGAGTTAGG CTCTGTCAGC CGCGGGTCCC TCGGGGGCCA AGGGCGAGGC<br>351 GCAGGCCGTC TGGCCGCAGG GAGAGGAACG GAGCGGGTCC CCCAGCGTGG TGCGCTTCCC TGAGCTGTGG<br>421 GACTTGCACC CGGGACTCGG CTCAAACACG C	chr14:64663221-64663671	AF221918.1	*Oryctolagus cuniculus* (AF221918)
*Equus caballus***	equCab3.0 (05/01/2018)	1 GGGTGGGGGA GAGTGGGTCT GGGCGGGGCG GCGGTCACGT TTTGT**CTAAC CCTAA**CTGAG CTGGGCGGAG<br>71 GCGCCGCGCT TTTGCTCCCC GCGCGCTGTT TTTCTCGCTG ACTTTCAGCG GGCGGAAAAG CCTCGGTCTA<br>141 CCGCCACTTA CCATCCAGTC TGGAGTAAAC AAAAAATGTC AGCCGCTGGC TCGCTCGCCC CTCCCGGGAC<br>211 CCTGCGACGG CTCGCCCGCC CAGCCCCCGC GCCCCGCCTG GAGGCCGCGG TCGGCCCGGG GCTTCTCCGG<br>281 AGGCGCCCAA TGCCGCCGCG AAGAGTTGGG CTCTGTCAGC CGCGGGTGCC TCGGGGGCCA GGGACGAGGC<br>351 TCTGGCCGCA GGGAGAGGAA CGGAGCGGGT CCCCGCGCGC GGTGCGCTTC CCTGAGCTGT GGGACGTGCA<br>421 CCCGGGACTC GGCTCAAACA CGT	chr19:12584771-12585213	AF221925.1	*Equus caballus* (AF221925)

Bos taurus	ARS-UCD1.2<br>(11/04/2018)	1 GGGTTGCGGA GGGTGGGCCC CGGGTTGGTG GCAGCCATTT CTCAT**CTAAC CCTAA**TTGAG ACAGGCGTAG<br>71 GCGCTGTGCT TTTGGTTACC GCGCGCTGTT TTTCTCGCTG ACTTTCAGCG GGCGGAAAAG CCTCGGCCTA<br>141 CCGCCATCCA CCATCCAGTC TGCAACAAAC AAAAAATGTC AGCCGCTGGC TCGCTCACCT CTCCCGGGAA<br>211 CCTGCGGTGG TCCGCCCGCC CAGCCCCAGT GCCCCGCCTG AGGCCGCGGT CGGCCCGGTG CTTCTCCGGA<br>281 GGTGTCCATT GCCGCCGTGA AGAGTTGGGC TCTGTCAGCC GCGGGTCGCT CGGTGGGCCG AGGCATGGCT<br>351 GTAACCGCAG GGAAAGGAAC GGAGTGGGGT CCCCGCGCGC GGTGCGCTTC CCTGAGCTGT GGGACTTGCA<br>421 CCCGGGACTC GGCTCAGACA TCC	chr1:97612791-97613232	NR_001576.1	*Bos taurus*<br>(NR_001576)
Sus scrofa	Sscrofa11.1<br>(07/02/2017)	1 GGGTTGCGGA GGGTGGGCCC AGGAGCGGTG GCGGCCATTT TTTAAGT**CTA ACCCTAA**CTG AAAGAGGCGT<br>71 AGGCGCTGCG CTTTTGCTTC ACGCGCGCTG TTTTTCTCGC TGACTTTCAG CGGGCGGAAA AGCCTCGGCC<br>141 TACCGCCATC CACCATCCAG TCTGAAACAA ACAAAAAATG TCAGCCTCTG GCTCGCTCAC TGCTCCCGGG<br>211 AACCTGCGGT GGTTCGCCCG CCCAGCCCCC GCGCCCCGCC TGAGGCCGCG GTCGGCCCGG GGCTTCTCCG<br>281 GAGGCGCCCA TTGCCGCCGC GAAGAGTTGG GCTCTGTCAG CCGCGGGTCC CTCGGGGGCC AAGGCGAGGC<br>351 TCTGACCGCA GGGAAAGGAA CGGAGTTGGT CCCCGCGCGC TGTGCGCTTC CCTAAGCTGT GGGCTGTGCA<br>421 CCTGGGACTC GGCTCAGACA CTT	chr13:108426066-108426508	AF221920.1	*Sus scrofa* (AF221920)
Felis catus	Felis_catus_9.0<br>(20/11/2017)	1 GGGTTGTGGA GGGTGGGCCT GGGAGGGGAA GCGGTCAGTT TTTGT**CTAAC CCTAA**CTGAG AAGGGCGTAG<br>71 GCGCCGCGCT TTTGTTTCCC GCACGCTGTT TTTTTCGCTG ACTTTCAGCG GGCGGAAAAG CCTCGGCCTA<br>141 CCGCCGTCCA CCGTACAGTT TGGAGCAAAC AAAAAATGTC AGCTGCTGAC TTGCTCGCCC CTCCCAGGAC<br>211 CCTGCGGTGG CTCGCCTCCT TAGCCCCCGC GTCCCGCCTA GAGGCCGCGG TCGGCCCGGG GCTTCTCCGG<br>281 AGGCACCCAT TGCCGTCGCG AAGAGTTGGG CTCTGTCAGC CGCGGGTCCC TTGGGGGGGC CGAGGGCGAG<br>351 GCTCTGACCG CAGGGAGAGA AACGGGAGCA GGTCCCCGCG CGCGGTGCGC TTCCCTGAGC TGTGGGACTT<br>421 GCACCCGGGA CTGGGCTCAG ACACAT	chrC2:98452545-98452964	AF221939.1	*Felis catus* (AF22193)
Canis lupus familiaris	ROS_Cfam_1.0<br>(03/09/2020)	1 GGAGGGTGGG CCCGGGAGAG GCGGCGGCCG GCCTCCGT**CT AACCCTAA**CT GAGCAGGGCG TGGGCGCCGC<br>71 GCTTTTGTTC CCCGCGCGCT GTTTTTCTCG CTGACTTTCA GCGGGCGGAA AAGCCTCGGC CTACCGCCGT<br>141 CCACCGTCCG GGTCTGCAGC CAACAACAAA AAATGTCAGC CGCTGGCTCG CTCGCCCCTC CCGGGAGCCT<br>211 GCGGCCGCTC GCCCGCTCGG CCCCCCGCGT CCCGCCCGGA GGCCGCGGCC GGCCCGGGGC TTCTCCGGAG<br>281 GCGCCCACTG CCGTCGCGAA GAGTTGGGCT CTGTCAGCCG CGGGCCGCCT GGGGGCCGCA GGGCGGGGCT<br>351 CGGGCCGCAG GGAGAGCAAC GGAGCGGGTC CCCGCGCGCG GTGCGTCTCC CTGAGCTGTG GGACTCGCAC<br>421 CCGGGACCGG GCTCGCACAC	chr34:34447071-34447510	No	*Felis catus*<br>(AF221939)
Loxodonta africana	LoxAfr3.0<br>(15/07/2009)	1 TGCGGTGGGT TGAGGAGGGT ACGCCCGGGA GGGCGGTGGT CTGTTCTGTT **CTAACCCTAA** CTGATAAGGG<br>71 CGTAGGCGCC GTGCTTTTGT TCCCCGCGCG TTGTTTTTCT CGCTGACTTT CAGCGGGCGG GAAAAGCCTC<br>141 GGTCTACCGC CGTCTACCGA TAGCCTGGAG CAAACAAAAA AATGTCAGCC GCCGGCCGCT CGCCCCTCCC<br>211 GGGAACCTGC AGTGGCTCGC CCGCCCAGCC CCGCTCCCCG CCTGGAGGCC GCGGTCGGCC TGGGGCTTCT<br>281 CCGGAGGTTC CCGCTGCCGC CGCGAAGAGT TGGGCTCTGT CAGCCGCGGG TCCCGCGGGA ACCAAGGGCG<br>351 AGGCTGGGGC CTCCTGAACG CAGGGAGAGA AACGGAGCGG TTCCCCGCGT GCGTGCGCTT CCCTGAGTTG<br>421 TGGGATGTGC GCTCGGGGCT CAGCTCCGAC AGGT	Scaffold_42:10605978-10606432	No	*Elephas maximus*<br>(AF221932)
Tapirus indicus	TapInd_v1_BIUU<br>(15/01/2019)	1 GGGTGGTGGA GAGTGGGTCC GGGAGGGCTG GCGGTCATGC TTTGT**CTAAC CCTAA**CTGAG CCGGGCGTAG<br>71 GCGCCGCGCT TTTGCTCCCC GCGCGCTGTT TTTCTCGCTG ACTTTCAGCG GGCGGAAAAG CCTCGGCCTA<br>141 CCGCCACTTA CCATCCAGTC TGGAACAAAC AAAAAATGTC AGCCGCTGGC TCGCTCGCTC TTCCCGGGAT<br>211 CCTGCGGTGG CTCGCCCGCC CGGCCCCCGC GCCCCGCCTG GAGGCCGCGG TCGGCCCGGG GCTTCTTCGG<br>281 AGGCACCCAA TGCCGCCGCG AAGAGTTGGG CTCTGTCAGC CGCGGGTGCC TCGGGGGCCA AGGGCGAGGC<br>351 TCTGGCCGCA GGGAGAGGAA CGGAGCGGGT CCCCGCGCGC GGTGCGCTTC CCTGAGCTGT GGGACGTGCA<br>421 CCCGGGACTC GGCTCAAACA CGT	Scaffold15412:32659-33101	No	*Equus caballus*<br>(AF221925)

Sarcophilus harrisii	mSarHar1.11<br>(07/11/2019)	1 GTGCGGACGG GCTGCGTGGG CGGGTCGGTC CGTCCTGGCA CAT**CTAACCC TAA**ATTCGCG CTGGTTGAAG<br>71 TGGCTTCTCC TGGGCGATCG CTCGCTGTTT TTGTGGCTGG CTTTCAGCGG GCTGGAGGAG CCGGGAGCGG<br>141 AGGCGGAGGA CCGAGCCAAA AACGTCAGCC GAGGCCGGGC GGCCCCGCTC GTGGCCCGTC CGCACTGTCC<br>211 GGGCCAGGCC CCGGCCCGAA CCCCCTGGAG ACGCCTCCCG CAAGGTCTGC GCTGCGCCGC GGAGCCCCGG<br>281 CTGCCCACGT AGAGGCGGCG GCCGTCTCGG GGCCCCCGAG TGCGGGGCGC CCACTGCGGC CGCCAAGAGC<br>351 TCGTCTCTGT CAGCCTCGGG TGCACGTGGG CCCGCGGTCG AGCCCTAAGG CCGGGCGTGG GGCGGGGGCC<br>421 GCAGGGAGAG TAACCGTGAG CCGGCGCCCA GCCTTCAGGG CGCTCCCTCG AGCTATGGGA GCTGCCCCCG<br>491 GGCACGGCTC AGACACCC	chr3:27711683-27712190	No	*Dasyurus hallucatus*<br>(AF221919)
Macropus eugenii	Meug_1.1<br>(06/11/2009)	1 CGGGCGGTCG GGCTGCAGCG GCGGGTCCCC TGGTCCTGCC CCTT**CTAACC CTAA**ATGCGC TCTGGGTGAA<br>71 GTGGCTCCTC CCGGGGGGTG CCCGCTGTTT TGGTGGCTGG CTTTCTGCGG GCAGGAGGAG CCGGGAGCGG<br>141 AGCGGAGACA GGAGGAAAAA ACGTCAGCCG AGGCTGGGCG GCCCCGCACG TGGCCCGTCC GCCCCGTCCA<br>211 GGTCCACCCC GGCCCGAACC CCCGGAGGAC GTCCCCCGCG GGGCCTGTGC TCGCCCACGG AGCCCCGGCC<br>281 GCCCAGGAGC AGAGGCGGCG GCCGTCTCGG GGCCCCTGCG TGCGGGGGCA CCCACTGAGG CCGCCAAGAG<br>351 CTGGGCTCTG TCAGCCTTGG GGTGCGCGTG GGGCGGTGGG CGAGCCCCAA GGCCGGGCGG GGGGCGGAGG<br>421 CCTCAGGGAG AGTAAGCGTG AGCGGCGCCC CGACTGCAGG GCACTCCCTC GAGCCATGGG AGCTGCCCCC<br>491 GGGCTCGGCT CAGACACCC	Scaffold_317:57-565	No	*Dasyurus hallucatus*<br>(AF221919)
Hirundo rustica	bHirRus1.pri.v2<br>(02/03/2021)	1 TGTGCCGGGG TGGGCAGGCC CTGCGGCCGA T**TAACCCTAA** TGGAGGCTGC CTCCCTCACA CCCGCCCGCT<br>71 GTTTTACTCG CTGACTTTCA GCGGGCAAAG GGAGCGGCCC AGGGAAAGGG GGGGATGGAG GGGGGGAAAA<br>141 AAAGTTCAGG CCTCAAAAAA AGCCAGCGAG GGGGATCCAC ACCCCGGCCC ACCCTGGGGT CCCTGTCCCT<br>211 CCTCCGCGAC CAGGGGTGGT CCCGCCCTCG CCCCTCGTGG AGGCCGCGGT CGGCAGGCCC AGCGCCACTA<br>281 CCGCCGCGAA GAGTTCGTCT CTGTCAGCCT CGGCGGCGGC CGGGATCGGA GGGCCGGCCC GCCCACCCAG<br>351 GGCAGCCGGG GGACCCCAGC AGAACAAAAC GGGGACGGCG CCGCGGGGCG CTGAGCACCC GTGGCTCCCA<br>421 AAGACGTGGA GTGCGCGGTC GGCGCCGCCC CGACACCC	chr10:21958559-21959016	No	*Anodorhynchus hyacinthinus*<br>(AF221924)
Gallus gallus	GRCg6a<br>(27/03/2018)	1 ACGCGTGGCG GGTGGAAGGC TCCGCTGTGC **CTAACCCTAA** TCGGGGGAAT TGATGGTGCT GTCGCCGCGC<br>71 TCCCTCCGCC CGCCCGCTGT TTTACTCGCT GACTTTCAGC GGGCGAGAGG AGCCGCCCCG GGGGGGAGGC<br>141 GGGCGGCGGG AGGGGGCCGG GGCGCCGCGG CGGTGGGGGT CGGGGGGGGG AGAGAAAGGG CCGAAAGGGG<br>211 CTCCGCGGCC AAAAAAACGT CAGCGAGGGG TCCGCTCGCC CCGATCCGCC CTGGGGTCCC CGCTCGCGTG<br>281 GCCGCGGTCG GCCGGCACCC GCCATTGCCG CCGCGAAGAG TTCGCCTCTG TCAGCCTCGG CGGCGCGCGG<br>351 GAGGTGCGGC GCGCGGCCCC GCGCCCCCAG CAGAGCAAAC GGGAGCGGCG CCCCCGGGGT AACCCCCGCG<br>421 CTCCCCTGCG CCGTGGGGCG CGCGGACGGC GTCGCTCCCA CACGC	chr9:20115803-20116267	NR_001594.1	*Gallus gallus*<br>(NR_001594)
Xenopus laevis	Xenopus_laevis_v2<br>(09/08/2016)	1 AATCAGCGTT TAAAGCTCAA TGTGGACGGA GGTCTCTGTT TCG**CTAACCC TAA**TACACTG GCTTCAGGGC<br>71 GATGGCTCTT CGCGGCGGTG CCTGTTGTTT TACTTGCTGA CTTTCAGCGG GCACGGAGAG CAAGCGTAGA<br>141 CGACGACTAA AAAACGTCAG CTGGGAGACT CCTCCGTTCG CACAGCCCGA CCTGCTCCAT TGCCCAAGAG<br>211 CCCCGGGTTT TCTCTGTGGA ATGTATCAGG CGCTCCCCGG TCTGTCTGTC TGGGGCCGCG GTCGGCATCA<br>281 TCTGCTGTCG CGAAGAGTTC GTCTCTGTTA GCCCTTGGGG GCCCTGGTGC GGAGTGGAGA GTCCGGGTCT<br>351 GGGGGGTCGG GAGAACAAAA GGGGGCGCGC TGGTGCTCAG GCTCAGTCAT GCTTTCCCTT AGTTGTGGGA<br>421 TATGCGTGTT CAGCCAGTCC CCGACATGT	chr9_10S:63011121-63011569	AF221908	*Xenopus laevis*<br>(AF221908)
Takifugu rubripes	FTakRub1.2<br>(05/06/2019)	1 ACGGAGTGTC TCTT**CTAACC CTAA**ATTATC TCGGCTCCCT GTACTCGGCG TTTGCTGTTT TTGTCTGGCT<br>71 TTCAGGAAAC TACAGGGAGT CTTGGACGTC TCGGTTCACG GATCAAAAAA GCCAAGAAAA TCACTCCGTC<br>141 GCGTTCAGGT CCCCCCCAGG AACACCGAGA GCCCTGTTGT GGTCAGTCCG GCTGCCGCAA AGAGTTGGTC<br>211 TCTGCTGCTC CGGTGTCTTG GCGGGATCAC CAAGGACTGA ACAGAGAAAC AGTGAGCGTG GTGACTCTAC<br>281 CGGTTCTTCC AATGTCCCGG TTAGGATACC CAAGCTCAAA CACTC	chr5:12035678-12036002	EF569638.1	*Takifugu rubripes*<br>(EF569638)

Scyliorhinus torazame	Storazame_v1.0 (02/08/2018)	1 GGGGCGGTGT CGTTTTTTA**C TAACCCTAA**T TTGGTGTAAG TGGTTGGGCC TCCCTCACGC TGCGGGAAAC<br>71 CTCGCTGTTT TCTTGGCTAA CTTTCAGCGA GAAGGTGAGG AGGGTTGGCG GCAAAAAGGT TGGGCAGCAA<br>141 CGGCGAGCGA ACAAAAAAAG TTAGAGCCGA GGCGCCGTTT TTTCCCCCCC CGCGGCCTGG TCTGAGGGTA<br>211 ACAAGGCCCT GCGGGAGAGG CGCACAAGCA ATGGGGGCCC CGGTCCGGCT AACGGTAGAG GAGGCTGGCC<br>281 CTCATCGCTT TCCAGGCCCA GCCACTCCGG GCCGCGGTCA GCCCCGGCTT CCCCCTCGGG GGGGGGCCTA<br>351 ATGCTGCCGC GAAGAGTTCG GCTCTGTCAG CCCGGCCGGG CCAGGGTGGA GGCCGGCACA GAGGGACACA<br>421 GGACCGGGGC GCCGGGGATA GCAACTTCCC GCAGGGGACA TTAAATATCA GTGACCAGTG GCTTCCATGT<br>491 GGCTGCATCT CCCGAAGCAA TGGGAGCTGC AAGGCTGCCC TGGTCATACA	scf_scyto00003180:22255-22794	No	*Rhizoprionodon porosus* (AF221915)
Petromyzon marinus	kPetMar1.pri (24/02/2020)	1 CTCCCCGTTG GCCCGCGAGC TCTTGCTCGT T**CTAACCCTA A**TTGTTTCCT TTGGCCTCTC TCAATTGTTT<br>71 GCTGAGTTTT GGCCGAGGTC CAGCAAAACG AGAGAGGCGT TCCACGGGCG TTGGTTTAAA ACTTGACAAA<br>141 CCTCGGCTCC CACCAATCGG GGAGTTAGGC CTCCGCAATC GTGTTACCCC CCCCCGCCAA CCTCGTCGGC<br>211 TCGCTTCCCA CGACCTCGCC TGGCCCCGGC GGTCCCGGCC TTCGTGCCCT CGCCGCCGGG AGGAGTTCGC<br>281 CTCCGCCGCC TCGCGAGCCG AGCCGTGGGG GGCCCACGAT GCGAGAGTAA CGAGAGGGTT CGGGTCTCCG<br>351 CTGGGCTTCC CCCTGATTGG TGGGGATTGC ACTTCTGGCC GCCTCTTACA TGT	chr27:7475598-7476001	BK012865.1	*Petromyzon marinus* (BK012865)
Lethenteron camtschaticum	LetJap1.0 (13/09/2013)	1 CACTCCCCGT TGGCCTCCGA GCTTCTTGCT CGTT**CTAACC CTAA**TTGTTT CATTTGGCCT CTTCTCAATA<br>71 TTTGCTGAGT TTTGGCCGAG GTCCAGCAAA AACGAGAGAG GCGTTCCACG GACGTTGGTT TTAAAACTTG<br>141 ACAAACCTCG GCTCCCACCA TTCGGGGAGT AAGGCCTCCG CAATCGGGTG CGAGTTTCCC CCCCCAAACC<br>211 TCATCCCACG GCCTCGCCTG GCCCCGGCGT GCCCGGCCTT CGTGCCCTCG CCGCCGGGAG GAGTTCGTCT<br>281 CCGCCGCCAC GCGAGCCCTG GGGGGGGGGG CCCCCACGAT GCGAGAGTAA CGAGAGGGTT CGGGTCTACG<br>351 ATGGGCTTCC CCCTTGATTG GTGGGGATTG CACCTCTGGC CGCCTCTTAC ATTT	contig065527:144-547	BK012866.1	*Lethenteron camtschaticum* (BK012866)
Eptatretus burgeri	Eburgeri_3.2 (24/10/2017)	1 CACTCCCCGT TGGCCTCCGA GCTTCTTGCT CGTT**CTAACC CTAA**TTGTTT CATTTGGCCT CTTCTCAATA<br>71 TTTGCTGAGT TTTGGCCGAG GTCCAGCAAA AACGAGAGAG GCGTTCCACG GACGTTGGTT TTAAAACTTG<br>141 ACAAACCTCG GCTCCCACCA TTCGGGGAGT AAGGCCTCCG CAATCGGGTG CGAGTTTCCC CCCCCAAACC<br>211 TCATCCCACG GCCTCGCCTG GCCCCGGCGT GCCCGGCCTT CGTGCCCTCG CCGCCGGGAG GAGTTCGTCT<br>281 CCGCCGCCAC GCGAGCCCTG GGGGGGGGGG CCCCCACGAT GCGAGAGTAA CGAGAGGGTT CGGGTCTACG<br>351 ATGGGCTTCC CCCTTGATTG GTGGGGATTG CACCTCTGGC CGCCTCTTAC ATTT	chr27:7475598-7476001	BK012871.1	*Eptatretus burgeri* (BK012871)

Table S2. Vertebrate TERC-ITS loci used in this study.

*Loci previously identified [34,35].

Locus number	Species	TERC-like sequence: Insertion site	TERC-like sequence: Position within TERC RNA sequence	TERC-like sequence: Length (bp) (Number of mismatches)	TERC-like sequence: TERC region	ITS length (bp) (Number of mismatches)	Locus organization	Insertion site modification	TERC used as reference for secondary structure analysis
1	*Homo sapiens*	chr6:33092493-33092517	8-32	25 (3)	Pseudoknot domain, template	6 (0)	B	Insertion + Deletion	*Homo sapiens*
	Pan troglodytes	chr6:32607352-32607376	8-32	25 (5)	Pseudoknot domain, template	6 (0)			
	Gorilla gorilla gorilla	chr6:33158042-33158066	8-32	25 (5)	Pseudoknot domain, template	6 (0)			
	Pongo pygmaeus abelii	chr6:33417464-33417488	8-32	25 (3)	Pseudoknot domain, template	6 (1)			
	Macaca mulatta	chr4:136413412-136413436	8-32	25 (3)	Pseudoknot domain, template	6 (0)			
	Callithrix jacchus	chr4:38087779-38087802	8-31	24 (3)	Pseudoknot domain, template	6 (0)			
	Saimiri boliviensis	scaffold00008:30970797-30970821	8-32	25 (4)	Pseudoknot domain, template	6 (0)			
2	*Homo sapiens*	chr18:73830841-73830884	15-45	31 (10)	Pseudoknot domain, template	14 (0)	B	Not Informative	
	Pan troglodytes	chr18:68063103-68063146	15-45	31 (10)	Pseudoknot domain, template	14 (0)			
	Gorilla gorilla gorilla	chr18:67137871-67137914	15-45	31 (10)	Pseudoknot domain, template	14 (1)			
	Pongo pygmaeus abelii	chr18:69355462-69355505	15-45	31 (11)	Pseudoknot domain, template	14 (1)			
	Macaca mulatta	chr18:6421937-6421980	15-45	31 (10)	Pseudoknot domain, template	14 (0)			
	Callithrix jacchus	chr13:112407855-112407879	15-45	31 (15)	Pseudoknot domain, template	14 (3)			
3	*Microcebus murinus*	chr3:9808477-9808511	120-154	35 (2)	Pseudoknot domain, J2a/3	43 (1)	A	Insertion + Deletion	
4	*Microcebus murinus*	chr4:11270478-11270504	142-168	27 (0)	Pseudoknot domain, J2a/3	43 (0)	A	Insertion + Deletion	
5	*Microcebus murinus*	chr6:96205947-96205978	124-154	31 (2)	Pseudoknot domain, J2a/3	25 (0)	A	Not Informative	
6	*Microcebus murinus*	chr14:66686502-66686535	127-161	35 (4)	Pseudoknot domain, J2a/3	32 (1)	A	Insertion + Deletion	

7	*Microcebus murinus*	chr32:2429016-2429039	129-156	28 (2)	Pseudoknot domain, J2a/3	38 (6)	A	Insertion + Deletion	
8	*Mus musculus**	chr1:46771696-46771729	341-374	31 (5)	ScaRNA Domain	57 (0)	A	Deletion	
9	*Mus musculus**	chr1:69071165-69071214	346-395	50 (2)	ScaRNA Domain	139 (16)	A	Insertion	
10	*Mus musculus**	chr4:119841389-119841433	351-392	42 (1)	ScaRNA Domain	53 (3)	A	Deletion	
11	*Mus musculus**	chr5:25644087-25644123	357-393	37 (3)	ScaRNA Domain	21 (2)	A	No modification	
12	*Mus musculus**	chr6:67988979-67989022	351-394	44 (1)	ScaRNA Domain	93 (0)	A	Not Informative	
13	*Mus musculus**	chr8:23257793-23257830	351-388	38 (3)	ScaRNA Domain	213 (32)	A	Not Informative	
	*Rattus norvegicus**	chr16:69254731-69254768	351-388	38 (4)	ScaRNA Domain	58 (8)			
14	*Mus musculus**	chr9:47869714-47869777	314-373	60 (1)	ScaRNA Domain	68 (1)	A	No modification	
15	*Mus musculus**	chr10:20335691-20335788	289-388	98 (10)	ScaRNA Domain	108 (4)	A	No modification	
16	*Mus musculus**	chr10:58382382-58382499	271-388	118 (11)	ScaRNA Domain	27 (1)	A	Deletion	
17	*Mus musculus**	chr11:86905013-86905050	343-381	38 (4)	ScaRNA Domain	55 (6)	A	Not Informative	
18	*Mus musculus*	chr12:7976282-7976348	316-384	68 (12)	ScaRNA Domain	77 (3)	A	Not Informative	*Mus musculus musculus*
	Rattus norvegicus	chr6:30794373-30794428	319-375	56 (8)	ScaRNA Domain	259 (42)			
19	*Mus musculus**	chr12:111293353-111293433	308-388	81 (5)	ScaRNA Domain	13 (0)	A	Deletion	
20	*Mus musculus**	chr13:3641558-3641615	331-384	54 (7)	ScaRNA Domain	22 (2)	A	Not Informative	
	*Rattus norvegicus**	chr17:66620909-66620960	331-384	53 (9)	ScaRNA Domain	22 (2)			
21	*Mus musculus*	chr17:77320142-77320174	360-392	33 (2)	ScaRNA Domain	58 (4)	A	Insertion + Deletion	
22	*Mus musculus**	chrX:65734790-65734861	322-395	74 (3)	ScaRNA Domain	23 (1)	A	Deletion	
23	*Mus musculus**	chrX:99124249-99124300	328-377	52 (9)	ScaRNA Domain	25 (1)	A	Not Informative	
	*Rattus norvegicus**	chrX:65181452-65181501	328-377	52 (9)	ScaRNA Domain	43 (4)			

24	*Cricetulus griseus*	scaffold364:1907954-1908002	332-380	49 (5)	ScaRNA Domain	60 (9)	A	Insertion	
25	*Cricetulus griseus*	scaffold477:2816499-2816590	283-374	92 (14)	ScaRNA Domain	205 (30)	A	No modification	
26	*Cricetulus griseus*	scaffold628:177514-177557	336-378	43 (9)	ScaRNA Domain	36 (3)	A	Not Informative	
27	*Cricetulus griseus*	scaffold2535:334947-335044	283-380	98 (9)	ScaRNA Domain	113 (14)	A	Insertion + Deletion	
28	*Oryctolagus cuniculus*	chr20:24654676-24654712	245-281	37 (2)	CR4/CR5 domain	66 (1)	A	Insertion	*Equus caballus*
29	*Oryctolagus cuniculus*	chrUn0052:1868849-1868866	148-165	18 (0)	Pseudoknot domain, J2a/3	41 (1)	A	Insertion + Deletion	
30	*Bos taurus*	chr12:22246021-22246046	125-150	26 (0)	Pseudoknot domain, J2a/3	24 (1)	A	Insertion	
31	*Bos taurus*	chr20:7319073-7319096	124-147	24 (0)	Pseudoknot domain, J2a/3	23 (0)	A	Deletion	
32	*Sus scrofa*	chr7:70772067-70772095	139-167	29 (1)	Pseudoknot domain, J2a/3	40 (7)	A	Insertion + Deletion	
33	*Equus caballus**	chr19:10034300-10034316	148-164	17 (0)	Pseudoknot domain, J2a/3	35 (0)	A	No modification	
34	*Tapirus indicus*	scaffold2309:127238-127269	128-160	33 (2)	Pseudoknot domain, J2a/3	26 (1)	A	Deletion	
35	*Loxodonta africana*	scaffold21:34372782-34372809	141-168	28 (0)	Pseudoknot domain, J2a/3	19 (1)	A	Insertion + Deletion	
36	*Macropus eugenii*	scaffold107834:53871-53949	424-502	79 (15)	ScaRNA Domain	12 (1)	A	Insertion + Deletion	*Dasyurus hallucatus*
37	*Hirundo rustica*	HRU5:20102963-20103004	106-146	42 (5)	Pseudoknot domain, J2a/3	10 (0)	A	Deletion	*Anodorhynchus hyacinthinus*
38	*Xenopus laevis*	chr2L:82565752-82565772	54-64	11 (0)	Pseudoknot domain, template	28 (0)	C	Insertion + Deletion	*Xenopus laevis*
39	*Takifugu rubripes*	chr18:10170798-10170815	7-14	8 (0)	Pseudoknot domain, template	46 (1)	B	Not Informative	*Takifugu rubripes*
40	*Scyliorhinus torazame*	scf_scyto00000011:2181744-2181774	233-263	31 (4)	Region P4-P4.1	30 (1)	A	Not Informative	*Rhizoprionodon porosus*
41	*Scyliorhinus torazame*	scf_scyto00006538:68375-68401	4-19	16 (1)	Pseudoknot domain, template	27 (0)	B	Not Informative	

```
(a) HSA18:73830841 (locus #2)
                3'-CAAUCCC-AAUCUGUUUUUUACCGGUGGUG--GGGAGGGUCCGGG-5'
HSA   CTAAAAAGACCAAATGTAGGAGGGTTAGGG-TTAGACAAActATaGtgcCCACggCtgTCCCAGGCCCCGGGCTATCTCCTACCCCA
PTR   CTAAAAAGACCAAATGTAGGAGGGTTAGGG-TTAGACAAActATaGtgcCCACggCtgTCCCAGGCCCCGGGCTATCTCCTAtCCCA
GGG   CTAAAAAGACCAAATGTAGGAcGGTTAGGG-TTAGACAAActATaGtgcCCACtgCtgTCCCAGGCCCCGGGCTATCTCCTACCCCA
PPA   CTAAAAAGACCAAATGTAGGAGGGTTAGGG-TcAGACAAGctATaGtgcCCACtgCtgTCCCAGGCCCCGGaCTATCTCCTACCCCA
MMU   CTAAAAAGACCAAATGTAGGAGGGTTAGGG-TTAGACAAGctATaGCgcCCACtgCtgTCCCAGGCCCtaGGCTATCTCCTACCCCA
CJA   CTAAAgAGACtggATGTAGGAGGGTT-GGGaTT-GACtAGctGTGGtgcCCACggCtgTCCCAGGtCCCaGGCTGcCTCCcAC-CCA

(b) MMR3:9808477 (locus #3)
               5'-GGCGGAAAAGCCUUGGCCUACUGCCGUCCACAGUC-3'
MMR   AGCCCATCAGGTG----GAAGAGGCGGAAAAGCCTTGGCCTgCTGCGGTCCACtGTCCGG (TTAGGG)6 TTAGGTCATTAGACCG
OGA   AatCtATCgGGTGacat-------------------------------------------   ------   -----TaAcTAGttCa

(c) MMR4:11270478 (locus #4)
                       5'-GCCGUCCACAGUCUAGUUUGGAGCAAA-3'
MMR   TAAAACCAGAAGTCAG---     ---TGGCCGTCCACAGTCTAGTTTGGAGCAAAA (GGGTTA)7 ATGCACACAGTTCATGGGAG
OGA   TAAAgCtAGAAGTCAGctg.∫∫.tag------------------------------   ------   -----CACAaTTCATGGGAG
SBO   TgAAgCCAGAAGTCAGttg.∫∫.tgg------------------------------   ------   -----CACAGTTCATGGGAa
CJA   TgAAgCCAGAAGTCAGttg.∫∫.tgg------------------------------   ------   -----CACAGTTCATGaGAa
MMU   TgAAgCCAGAAGTCAGttg.∫∫.tgg------------------------------   ------   -----CACAGTTCATaGGAG
PPA   TgAAgCCAGAAGTCAGttg.∫∫.tgg------------------------------   ------   -----CACAGTTCATaGGAG
GGG   TgAAgCCAGAAGTCAGttg.∫∫.tgg------------------------------   ------   -----CACAGTTCATacGAG
PTR   TgAAgC-AGAAGTCAGttg.∫∫.tgg------------------------------   ------   -----CACAGTTCATatGAG
HSA   agAAgC-AGAAGTCAGttg.∫∫.tgg------------------------------   ------   -----CACAGTTCATacGAG
                      |<-16 bp ->|

(d) MMR14:66686502 (locus #6)
                 5'-AAGCCUUGGCCUACUGCCGUCCACAGUCUAGUUUGG-3'
MMR   TCTTCAA-------TTA.∫∫.TTGAAGCCTTGGCCTACTGCCaTCtACAGTCcAGTcTGG (GGTTAG)4 AACCCTTTGCTCTGTA
OGA   TtTcgAAgccccct------------------------------------------------   ------   ------TTtgcCatac
SBO   aCTTtAAatcccct------------------------------------------------   ------   ------TTcCctTGTA
CJA   aCTTCAAtttccct------------------------------------------------   ------   ------TTcCctTGTA
MMU   cCTTtAAaacccct------------------------------------------------   ------   ------TTGCcaTGTA
PPA   ctTTCAAaacccct------------------------------------------------   ------   ------TTGCTaTGTA
GGG   TtTTCAAa-cccct------------------------------------------------   ------   ------TTGCTaTGTA
PTR   ctTTCAAaacccct------------------------------------------------   ------   ------TTGCTaTGTA
HSA   ctTTCAAaacccct------------------------------------------------   ------   ------TTGCTaTGTA
                    |<- 17bp ->|

(e) MMR32:2429016 (locus #7)
                 5'-GCCUUGG.∫∫.AGUCUAGUU-3'
MMR   TTCC----------CCA.∫∫.CATGCCTTGG.∫∫.AGTCcAGTTAGGGTTAcGGTTA (CCTAAC)3 CCTAAGCA.∫∫.CCAATAC
OGA   TcCCcct.∫∫.act---   ----------   ----------------------   ------   ---------   ---gTt-
SBO   TTCCcct.∫∫.acc---   ----------   ----------------------   ------   ---------   ---ATAC
CJA   TTCCctt.∫∫.acc---   ----------   ----------------------   ------   ---------   ---ATAC
MMU   cTCCcct.∫∫.gcc---   ----------   ----------------------   ------   ---------   ---ATAC
PPA   cTCCcct.∫∫.gcc---   ----------   ----------------------   ------   ---------   ---ATAC
GGG   cTCCcct.∫∫.gcc---   ----------   ----------------------   ------   ---------   ---ATAC
PTR   cTCCcct.∫∫.gcc---   ----------   ----------------------   ------   ---------   ---ATAC
HSA   cTCCcct.∫∫.gcc---   ----------   ----------------------   ------   ---------   ---ATAC
         |<-58 bp->|<-18 bp->|                                             |<-11 bp->|
```

Figure S1. TERC-ITS loci in primates. For each locus, the top row shows the sequence of the telomerase RNA domain (light blue) homologous to the inserted TERC-like fragment. Species names are indicated on the left. The first species is the one in which the TERC-ITS was initially identified and the following species are listed according to the evolutionary distance from the first species. Telomeric repeats in TTAGGG and CCCTAA orientation are indicated in red and orange, respectively. The TERC-like sequences homologous to a region of the Telomerase RNA are indicated in light blue and the sequence complementary to a region of the Telomerase RNA in dark blue. Modifications of the insertion sites are shown: underlined nucleotides indicate deletions and green nucleotides indicate random sequence insertions. Nucleotides at the 3' end of the TERC-like sequence in frame with the inserted telomeric repeats are boxed.

```
(a) Mus12:7976282 (locus #18)
                5'-AGGACAGGAAUG.∫∫.GCACCCGGAACUC-3'
Mus   AGTGCCTCCAAGGACAGGG-TG.∫∫.GCACCCaGAACTCTT (TTAGGG)_12 TTAGG------------------ACAGGGTCATG
RNO   gGTGCCTCCAAaacCgaGG-Ta.∫∫.GCACCCaGAACTCCT (TTAGGG)_42 TTAGGGTTAGATTTTGTCATCCT-CAtGGTCATG

(b) Mus17:77320142 (locus #21)
                 5'-GCUGUGGGAA.∫∫.UCGGUUCUCA-3'
Mus   CTGGATTTCCTGCTGTGGGAA.∫∫.TCtGTTCTCAAGGAAAAAAAAAAA (TTAGGG)_9 TTAACCT---     ---ATGTTCTCTAT
RNO   CTGGATTTtCT----------    ------------------------  -------   -------GGA.∫∫.TTGATGTTtCTCTAT
                                                                        |←62 bp→|

(c) CGR_scaffold477:2816499 (locus #25)
                         5'-GAGCCCCGGC.∫∫.AAGUCGGUUC-3'
CGR   CAGATATA.∫∫.TTTGTCAGAGCCCtGGC.∫∫.AAGTCGGTTCTAAAAATTcA (GGGTTA)_33 GGGCCTGTAT.∫∫.ACGATGAT

(d) CGR_scaffold628:177514 (locus #26)
             5'-CUUCCCUGAGUUG.∫∫.CUCGGUUCCCACA-3'
CGR   AACTCAGCTTCtCTGAGcTG.∫∫.CaCaGcTCaCACAGGa (GTTAGG)_5 GGGGGCTCAAACAACC.∫∫.TGAGGGAGCAGAAAGG

(e) CGR_scaffold2535:334947 (locus #27)
                                 5'-GAGCCCCGG.∫∫.UUCCCACAA-3'
CGR   TTTCTGCC---     ---CAG.∫∫.GTGGAGCCCCaG.∫∫.TTCCCAaAA (TTAGGG)_18 TTAGGATA.∫∫.ACAAGACAAATCA
MMM   TTTCTGCCaca.∫∫.tcc---    -------------    ----------  ------     ---------     ---AaACAAtTaA
RNO   TTTCTGCCaca.∫∫.cac---    -------------    ----------  ------     ---------     ---AaACAAtTtA
              |←60 bp→|←14 bp→|                                       |←19 bp→|

(f) OCU20:24654676 (locus #28)
                        5'-CCGCCUGAGGCCGCGGUCGGCCCGGGGCUUCUCCGGAGGU-3'
OCU   ACGTGTCTATTGATGTGACCGCCTGAGGCCtCGGTCGGCCCGGGGCTTCTCtGGAGGT (TAGGGT)_10 TGATTGTCTGCTGGCCT
LTI   cCtTGTCTATTGgT--------------------------------------------  ------     ---TctcCTGCTGGCCT

(g) OCU_Un0052:1868849 (locus #29)
                                          5'-UCCACCGUUCAUUUCGCAGU-3'
OCU   ATCCTTGATGCCACCT----     --TCT.∫∫.GTCTCCACCGTTCATTTCGCAGT (TAGGGT)_6 TAGAGACTATTAGAGTAAGA
LTI   gaCaTgGAatCAACCTgtg.∫∫.tag------------------------------    ------    ---taACTgTTAGAGTAgGc
                     |←58 bp→|← 38 bp →|

(h) BTA12:22246021 (locus #30)
                        5'-GAAAAGCCUCGGUCUACCGCCAUCCA-3'
BTA   TCAATCAATGGTAATACGAAAAGCCTCGGTCTACCGCCATCCAAAAAAA (GGGTTA)_4 TTT.∫∫.TCTCAGGAGAGTTATTTCTG
OAR   TCAATCAAcGGTAATgC----------------------------------  ------   ---     ---CAGGAGAGTTATTTCTG
SSC   c--------GGTAgcAC----------------------------------  ------   ---     ---CAGGAGgGTgATTcCTG
TTR   cCAAaaAATGGTAAcAC----------------------------------  ------   ---     ---CAGGAGgGTgATTTCTG
                                                                 |← 13 bp →|

(i) BTA20:7319073 (locus #31)
                      5'-GGAAAAGCCUCGGCCUACCGCCAU-3'
BTA   TGTCAGTA.∫∫.GTGGGAAAAGCCTCGGCCTACCGCCATAAAAGAA (TTAGGG)_3 TTAGG---     ---TCC.∫∫.AACTGAAT
OAR   TGTCAGTA.∫∫.GaGGGA------------------------------  ------    -----gac.∫∫.ggaTCC.∫∫.AACTGAAT
SSC   TGcCA---    -----------------------------------    ------    ---------    ------    ---TGAAT
TTR   TGTCA---    -----------------------------------    ------    ---------    ------    ---TGAAT
         |←62 bp→|                                              |←13 bp→|←141 bp→|

(j) SSC7:70772067 (locus #32)
                                    5'-CCUACCGCCAUCCACCAUCCAGUCU-GAA-3'
SSC   TTGAAAGAAA----------AGT.∫∫.CAACCTACtGCCATCCACCATCCAGTCTGGAAA (TTAGGG)_6 GTTAACAGTTAGACAA
BTA   TTGAAAGAAAtat.∫∫.tgc-----------------------------------------  ------    ------AtTTtGgCAA
OAR   cTGAAAGAAAtat.∫∫.tgc-----------------------------------------  ------    ------AtTTAGgCAA
               |←53 bp→|←328 bp→|
```

(k) TIN_scaffold2309:127238 (locus #34)

```
                           5'-AAGCCUCGGCCUACCGCCACUUACCAUCCAGUCU-3'
TIN  AAGGACCTGTTTGGGGGT------AAGCCTCGGCC-ACCGCCACTTACCgTCCAGTCTA (GGGTTA)_4 ACTAGCTCCATTTTTCC
CSS  AAGGACCTGTcTGGGG-Tgcttta-----------------------------------  ------   ACTAGCTCCATTTTcCC
ECA  AAGGACCTGTTTGGGGGTacttta-----------------------------------  ------   ACcAGCcCCATTTTTCC
                       |←6 bp→|
```

(l) LAF_scaffold21:34372782 (locus #35)

```
                   5'-GGCCUACGGCCGUCUACCGAUAGUCUGG-3'
LAF  TTCAGCCCAGAGGTTGGGCCTACGGCCGTCTACCGATAGTCTGG (TAGGGT)_3 TTAC----    ----CCCCACAGCACTAAGG
PCA  TTCAGCC-AGAGGTTGGG--------------------------  ------   ----gcag.ʃʃ.ggtaCCCtACAGCACcAAGG
                                                              |←— 14 bp ——→|
```

(m) MEU_scaffold107834:53871 (locus #36)

```
                            5'-CAGGGAGAGUAAGCG.ʃʃ.CCCCAGGCAUGGCUCA-3'
MEU  ACTC---------CTG.ʃʃ.CTACAGGGAGAGTAgcCa.ʃʃ.CCCCAGGCATGGCTCAATTA.ʃʃ.TTATTTAGaGTTAGGGAGTCT
SHA  ACTCacaaagata---    -------------------    --------------------    ----------------AaTtT
         |←—9 bp —→|←—12 bp ——→|                               |←—— 45 bp——→|
```

(n) HRU5:20102963 (locus #37)

```
              5'-GGCCCAGGGAAAGG.ʃʃ.G-GAAAAAAAGUU-3'
HRU  GAAGACATAGGCCCAGGGAAAGG.ʃʃ.GgGAA---AAGTTAAAAAAATAGGGTTAGG------------GTTATTTTCCCTGCTTCT
PDO  GAAGACATAG-------------    ------------------------------ctgcttctatgtGTTATTTTCCCTGCTTCT
CMO  GAAGACATAG-------------    ------------------------------ctgcttctatgtGTTATTTTCtCTGCTTCT
```

(o) XLA_2L:82565752 (locus #38)

```
                 3'-UUCGGUCACAUAAUCCCAAUC-5'
XLA  TGAATCAGAAACACTAAGCCAGTGTATTAGGGTTAGGGTTA (ACCCTA)_2 ATAC.ʃʃ.AAT---    ---ATATAAAATATAAT
XTR  TGAATCAGAAACAtTAAG-----------------------  ------   ----    ---ttc.ʃʃ.tcgAgATAcAcctatAT
                                                        |←—45 bp —→|←—13 bp ——→|
```

(p) TRU18:10170798 (locus #39)

```
                                               3'-AAUCCCAAUCUUCUCUGU-5'
TRU  GCTCAGCATCAACAACTGGACTGATGTGATCTGA (TTAGGG)_6 TTAGGGTTAGAAGAGACAGGAAGTGGACACAGACACGACTTT
```

(q) STO_scf_scyto00000011:2181744 (locus #40)

```
                5'-CACAAGCAAUGGGGGCCCCGGUCCGGCUAAC-3'
STO  ACCATTCTCCCGACACAAaCAATGGaGaCCCCtGTCCGGCTAACAGGAGGG (TTAGGG)_4 TTCTCCAGGAGAATCGGTGGACCGG
```

(r) STO_scf_scyto00006538:68375 (locus #41)

```
                                 3'-AAUCCCAAUCAUUUUUU-GCUGUGGCG-5'
STO  CCCCCCCCCCCCCC (TAGGGT)_2 TAGGGTTAGGGTTAGTAAAAAATCGACACCGCATCGGAAACTGGCCAGTGTCAAATTACATA
```

Figure S2. TERC-ITS loci in non-primate vertebrates. For each locus, the top row shows the sequence of the telomerase RNA domain (light blue) homologous to the inserted TERC-like fragment. Species names are indicated on the left. The first species is the one in which the TERC-ITS was initially identified and the following species are listed according to the evolutionary distance from the first species. Telomeric repeats in TTAGGG and CCCTAA orientation are indicated in red and orange, respectively. The TERC-like sequences homologous to a region of the Telomerase RNA are indicated in light blue and the sequence complementary to a region of the Telomerase RNA in dark blue. Modifications of the insertion sites are shown: underlined nucleotides indicate deletions and green nucleotides indicate random sequence insertions. Short microhomologies between the TERC-like sequence and the 3' end of the break site are indicated with nucleotides with yellow background. Nucleotides at the 3' end of the TERC-like sequence in frame with the inserted telomeric repeats are boxed.

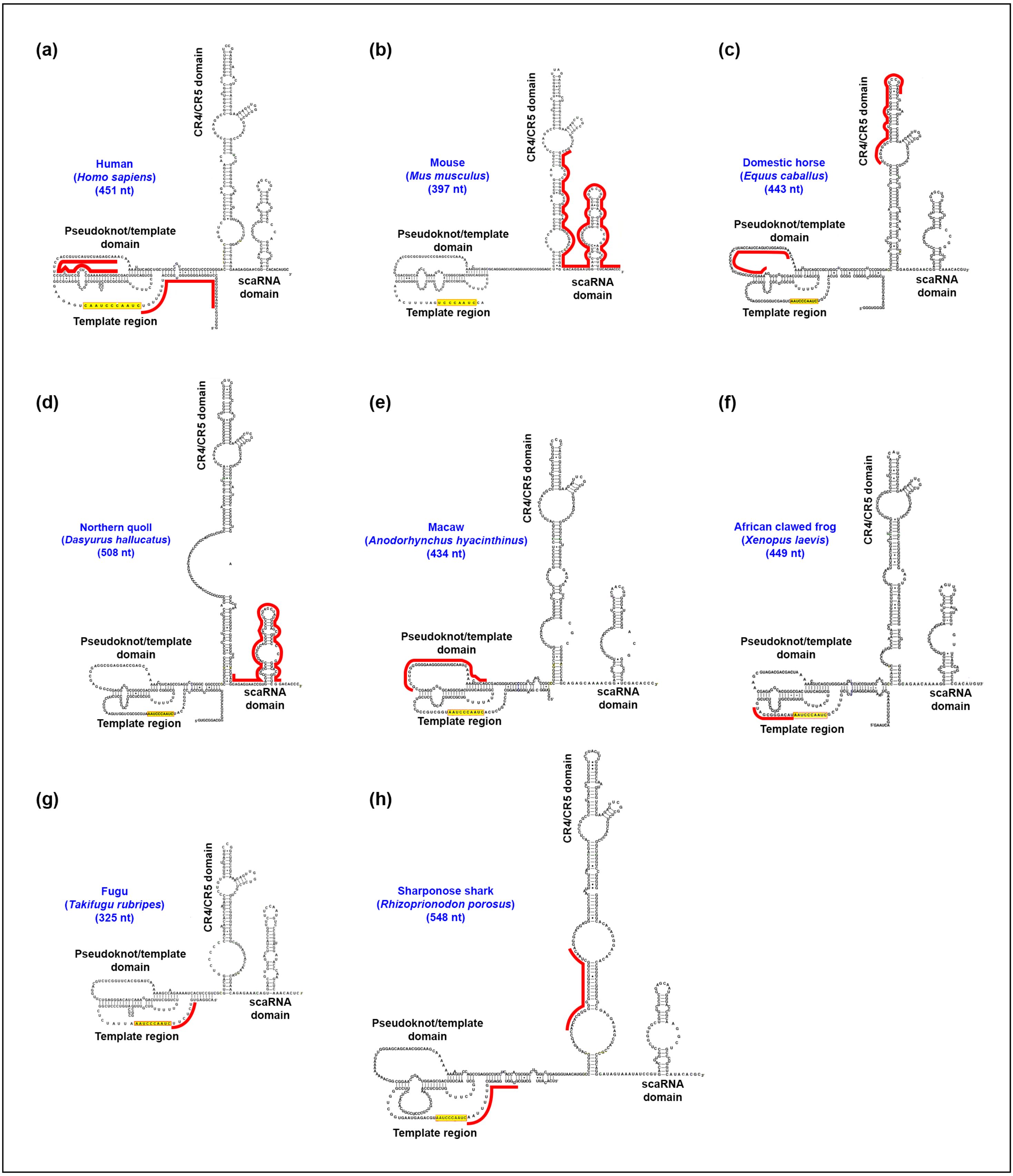

Figure S3. Regions of TERC-RNA homologous to TERC-ITS loci. The position of TERC-like sequences found at TERC-ITS loci as marked on the proposed secondary structures of the TERC RNA. Red lines cover the overall TERC regions retro-transcribed at relevant TERC-ITSs. The template region, which is retrotranscribed, by definition, at all loci, is not marked. **(a)** The seven primate TERC-ITSs are marked on the human TERC structure. The sequence immediately upstream of the telomeric repeat template is contained in two loci. The partially single stranded region J2a/3 in the pseudoknot is contained in five TERC-ITSs. **(b)** The TERC-like sequences from the 20 Rodentia loci are marked on the secondary structure of mouse TERC. All 20 TERC-like fragments from these species, in which the TERC RNA is lacking 43 nucleotides from the 5' end, derive from the scaRNA domain and the adjacent region, which are positioned at the 3' end of the TERC-RNA, far away from the pseudoknot. **(c)** The eight TERC-like sequences from non-primate and non-rodent mammals (rabbit, cow, pig, horse, tapir and elephant) are marked on the horse TERC RNA structure. Seven TERC-like fragments derive from the partially single stranded region J2a/3 within the pseudoknot. One TERC-like fragment (from rabbit locus 28) corresponds to a region of the CR4/CR5 domain. **(d-h)** TERC-like sequences from the loci found in a marsupial, a bird, a frog, a ray-finned fish and a shark are marked on the secondary structure of TERC from Northern quoll, macaw, African claw frog, tiger pufferfish and sharpnose shark, respectively. Each sequence marked on these structures derives from one TERC-ITS.
